# The *TYK2*-P1104A Autoimmune Protective Variant Limits Coordinate Signals Required to Generate Specialized T Cell Subsets

**DOI:** 10.3389/fimmu.2019.00044

**Published:** 2019-01-25

**Authors:** Jacquelyn A. Gorman, Christian Hundhausen, Mackenzie Kinsman, Tanvi Arkatkar, Eric J. Allenspach, Courtnee Clough, Samuel E. West, Kerri Thomas, Ahmet Eken, Socheath Khim, Malika Hale, Mohamed Oukka, Shaun W. Jackson, Karen Cerosaletti, Jane H. Buckner, David J. Rawlings

**Affiliations:** ^1^Center for Immunity and Immunotherapies, Seattle Children's Research Institute, Seattle, WA, United States; ^2^Translational Research Program, Benaroya Research Institute, Seattle, WA, United States; ^3^Department of Pediatrics, University of Washington, Seattle, WA, United States; ^4^Department of Immunology, University of Washington, Seattle, WA, United States

**Keywords:** TYK2, autoimmunity, lupus, Tfh, IL-12, IL-23, IFNAR, germinal center

## Abstract

TYK2 is a JAK family member that functions downstream of multiple cytokine receptors. Genome wide association studies have linked a SNP (rs34536443) within *TYK2* encoding a Proline to Alanine substitution at amino acid 1104, to protection from multiple autoimmune diseases including systemic lupus erythematosus (SLE) and multiple sclerosis (MS). The protective role of this SNP in autoimmune pathogenesis, however, remains incompletely understood. Here we found that T follicular helper (Tfh) cells, switched memory B cells, and IFNAR signaling were decreased in healthy individuals that expressed the protective variant *TYK2*^*A*1104^ (*TYK2*^*P*^). To study this variant *in vivo*, we developed a knock-in murine model of this allele. Murine *Tyk2*^*P*^ expressing T cells homozygous for the protective allele, but not cells heterozygous for this change, manifest decreased IL-12 receptor signaling, important for Tfh lineage commitment. Further, homozygous *Tyk2*^*P*^ T cells exhibited diminished *in vitro* Th1 skewing. Surprisingly, despite these signaling changes, *in vivo* formation of Tfh and GC B cells was unaffected in two models of T cell dependent immune responses and in two alternative SLE models. TYK2 is also activated downstream of IL-23 receptor engagement. Here, we found that *Tyk2*^*P*^ expressing T cells had reduced IL-23 dependent signaling as well as a diminished ability to skew toward Th17 *in vitro*. Consistent with these findings, homozygous, but not heterozygous, *Tyk2*^*P*^ mice were fully protected in a murine model of MS. Homozygous *Tyk2*^*P*^ mice had fewer infiltrating CD4^+^ T cells within the CNS. Most strikingly, homozygous mice had a decreased proportion of IL-17^+^/IFNγ^+^, double positive, pathogenic CD4^+^ T cells in both the draining lymph nodes (LN) and CNS. Thus, in an autoimmune model, such as EAE, impacted by both altered Th1 and Th17 signaling, the *Tyk2*^*P*^ allele can effectively shield animals from disease. Taken together, our findings suggest that TYK2^P^ diminishes IL-12, IL-23, and IFN I signaling and that its protective effect is most likely manifest in the setting of autoimmune triggers that concurrently dysregulate at least two of these important signaling cascades.

## Introduction

Systemic lupus erythematosus (SLE) comprises a group of heterogenous disorders classified under a broad clinical phenotype of systemic autoimmunity (1, 2). Loss of tolerance and sustained autoantibodies are key factors in the SLE pathogenesis (1). T cells play a critical role in SLE pathogenesis and previous work has identified alterations in CD4^+^ T cell subsets in patients with lupus (1). This reflects differentiation of naïve CD4^+^ T cells into alternative specialized T helper (Th) subtypes, including Th1, Th2, Th17, and T follicular helper (Tfh) cells. Differentiation is dependent on the cytokine milieu that the T cell encounters, and appropriate signaling through multiple cytokine pathways is required for lineage commitment. In SLE, heightened percentages of Tfh-like cells are present in both germinal centers and peripheral blood and correlate with serum autoantibody titers (3, 4). Tfh cells are key components of the adaptive immune response, providing the T cell help necessary for the development and maintenance of germinal center (GC) B cells and a robust antibody response (3, 5–7). Commitment to the Tfh lineage is driven by expression of transcription factor Bcl-6 expression (8). A number of cytokine signals have been implicated in the regulation of Bcl-6 expression, including IL-6, IL-21, IL-12, IL-2, IL-23, TGF-β, and IFN-γ through the Janus Kinase (JAK)-STAT pathways (9–13). Not surprisingly, dysregulation of these cytokine programs can contribute to disease through preferential expansion or depletion of particular Th lineages (3, 14).

Consistent with the altered T cell subsets observed in SLE, IL-12, and IL-23 levels have been found to be increased in SLE patients (3, 15, 16). Further, a positive correlation between levels of IL-12 and SLEDAI were seen in these lupus patients and active lupus nephritis had even higher levels of IL-12 compared to inactive SLE patients (15, 16). Type I interferons (IFN I) are also frequently upregulated in SLE subjects. IFN I impacts T cell subset commitment by promoting Bcl-6 expression, independent of STAT3 signaling and IL-21 production (17). However, IFN I has also been shown to be a corepressor of Tfh in the absence of STAT3 while augmenting interferon stimulated genes (ISGs) and Th1-like commitment (18). Given the complexity of T cell subset generation and the genetic heterogeneity of human autoimmunity, further work is needed to define the interplay of signals that control Tfh development and survival, and the role of T cell subsets in the pathogenesis of SLE and other autoimmune disorders.

TYK2 (non-receptor tyrosine-protein kinase), a member of the Janus Kinase (JAK) family, has been identified as a mediator in signaling cascades for IL-12, IL-23, IFN I, IL-6, IL-10, and IL-13 (19). The first human subject described with *TYK2* deficiency presented with hyper-IgE syndrome (HIES) (20). However, studies of additional *TYK2*-deficient subjects revealed specific alterations in cytokine signaling cascades without evidence for HIES (21, 22). Specifically, *TYK2*-deficient human T cells exhibited impaired responses to IL-12, IL-23, IFN-α, and IL-10 and these subjects presented with mycobacterial and viral infections (21). Consistent with these human data, *Tyk2*-deficient mice exhibit defective IL-12, IL-23, IFN I signaling and decreased Th1 *in vitro* skewing (23, 24). Further, TYK2 regulates early responses of IL-10 through Jak1-STAT3-SOCS3 signaling cascade (25). *Tyk2*^−/−^ mice are also more susceptible to vesicular stomatitis virus (VSV), and murine cytomegalovirus (MCMV) but, intriguingly, are protected from experimental autoimmune encephalomyelitis (EAE) (19, 23, 26, 27).

Genome wide association studies (GWAS) have identified a single nucleotide polymorphism (SNP; rs34536443) in the *TYK2* gene associated with several autoimmune diseases (28–33). This SNP results in a proline to alanine substitution at amino acid 1,104 in the kinase domain of the protein (P1104A; A1104 referred to hereafter as *TYK2*^*P*^) (31). Strikingly, the *TYK2*^*P*^ variant has been associated with protection from multiple autoimmune diseases including: SLE, type 1 diabetes (T1D), multiple sclerosis (MS), rheumatoid arthritis, psoriasis, Crohn's disease, inflammatory bowel disease, and ulcerative colitis (28–34). Early studies suggested that *TYK2*^*P*^ was a hypomorphic allele (35, 36). However, these studies reported conflicting results using alternative cell lineages suggesting that the signaling activity of the variant might depend on context and cell type (35, 36). More recent work has shown that *TYK2*^P^ leads to hypomorphic signaling including reduced IFN I responses in all cell types and reduced IL-12/IL-23 signaling in human and murine T cells (33). The precise role(s) for *TYK2*^*P*^ in altering autoimmune pathogenesis, however, remains poorly elucidated.

In the current study, we utilized cells from healthy human subjects with the variant and knock-in mice to assess the impact of *TYK2*^*P*^ on T cell subsets and cytokine signaling and on normal and autoimmune responses *in vivo*. First, we demonstrate that healthy individuals with the protective variant exhibit decreased IFN I signaling and have a decreased frequency of circulating Tfh cells and switched memory B cells. We established a knock-in murine model of this allele and show that homozygous *Tyk2*^*P*^ T cells exhibit decreased IL-12 receptor signaling and diminished *in vitro* Th1 skewing. Surprisingly, *in vivo* formation of Tfh and GC B cells was unaffected by *Tyk2*^*P*^ expression in alternative murine models of T cell dependent immune responses. Further, expression of the protective variant did not protect against murine lupus in alternative murine SLE models. Additionally, we found that *Tyk2*^*P*^ expressing T cells had reduced IL-23 dependent signaling and diminished ability to skew toward Th17 *in vitro*. Unlike lupus murine models, homozygous *Tyk2*^*P*^ mice were fully protected from EAE, and infiltrating CD4^+^ T cells within the CNS. Moreover, homozygous variant mice had a markedly decreased population of pathogenic IL-17^+^/IFNγ^+^ CD4^+^ T cells in both the draining lymph nodes (LN) and CNS. Thus, our data suggest that TYK2^P^ reduces IFN I, IL-12, and IL-23 signaling in T cells, and that only when autoimmune disease synchronously dysregulates multiple cytokine signaling programs will the protective phenotype be observed.

## Materials and Methods

### Human Samples and Genotyping

Cryopreserved PBMCs were obtained from adult participants in the Benaroya Research Institute (BRI) Immune Mediated Diseases Registry and Repository. Subjects were selected based on *TYK*2 genotype and the absence of autoimmune disease or any family history of autoimmunity. Study groups were designated as follows: subjects homozygous for the non-protective (NP) allele “C” at rs34536443: “NP/NP”; subjects homozygous for the protective (P) allele “G” at rs34536443: “P/P”, and heterozygous subjects: “NP/P”. *TYK2* SNP rs2304256 was held constant “C/A” as far as possible (all NP/NP and NP/P subjects). The “P/P” group was homozygous “A/A” at rs2304256 in all cases. Subjects were age matched (mean age: NP/NP group, 37.7 ± 12.6 years; NP/P group, 37.7 ± 14.3 years; P/P group, 45.3 ± 18.1 years) and sex matched as far as possible (NP/NP group, 21 males and 20 females; NP/P group, 15 males and 17 females; P/P group 3 male and 1 female). All experiments were performed in a blinded manner with respect to *TYK2* genotype. Genomic DNA was genotyped for the *TYK2* SNPs rs34536443 (C/G) (P1104A) and rs2304256 (C/A) (V362F) using a Taqman SNP genotyping assay (Applied Biosciences) or were genotyped using the Illumina ImmunoChip by the University of Virginia Center for Public Health Genomics. The Taqman genotyping assay was validated using HapMap DNAs of known genotype, and controls of each genotype were included in every genotyping experiment. Results were checked for adherence to Hardy-Weinberg equilibrium. The research protocols were approved by the Institutional Review Board at BRI (#07109-148).

### Mice

A construct designed to generate a P1124A mutation in exon 21 of *Tyk2* by homologous recombination in C57BL/6J mice was generated and injected by Biocytogen as previously described (37). After successful recombination, two FRT sequences with a neomycin-resistance selection cassette were inserted into intron 21. To create lineage-specific deletion, *loxP* sites were also present in intron 19 and 21. C57BL/6J embryonic stem (ES) cells had the introduction of the construct and clones were obtained by limited dilution. G418 selection was used to select clones. Clones that contained successful integration of the knock-in template into the locus were confirmed by Southern blot and PCR analysis of genomic DNA. Successfully targeted clones were injected into BALB/c blastocysts and were subsequently transferred into pseudopregnant females. One clone gave rise to a line with germline transmission of the allele. The mutation was confirmed by sequencing of Exon 21 (Supplementary Figure 1), and PCR was used to genotype all litters (using the following primers: 5′-CCACTCCTAACCTTGTAGAGCAC-3′ and 5′-AACGCAAATCTCTACAACAGTGG-3′). Mice were crossed with B6.Cg-Tg(ACTFLPe)9205Dym/J (Jackson Laboratory) mice to delete the neomycin-resistance selection cassette. In Th1 skewing assays, *Tyk2* knockout mice were created by Dr. Mathias Müller and were kindly provided by Dr. George Yap (23). *Tyk2* knockout mice were also generated by crossing *TYK*2^*P*^ mice with B6.C-Tg(CMV-cre)1Cgn/J (Jackson Laboratory) strain to make a global knockout of *Tyk2*. Deletion was confirmed by sequencing *loxP* sites (Supplementary Figure 1), and PCR was used to genotype all litters (using the following primers: 5′-CCACTCCTAACCTTGTAGAGCAC-3′ and 5′-CCTCCCTGTGTGTGATGTGG-3′). WAS–/– mice are on a C57BL/6J background (38). All strains were maintained in a specific-pathogen-free facility, and studies were performed in accordance with procedures approved by the Institutional Animal Care and Use Committees of Seattle Children's Research Institute.

### *In vitro* Stimulation and Th Skewing Assays

For IL-12 signaling, thawed PBMCs were washed and resuspended in complete medium (RPMI, 10% human serum, 1% PenStrep) at 4 × 10^6^ cells/ml. Cells were activated with anti-CD3/CD28 Dynabeads (ThermoFisher) at a bead to cell ratio of 1:10 for 72 h. Following removal of the magnetic beads, cells were rested in *X-vivo* 15 medium (Lonza) for 2 h, washed with PBS and stimulated with 2.5 ng/ml of recombinant human IL-12 (BD Pharmingen) for 30 min. For IFN-α signaling, thawed PBMCs were washed and rested in *X-vivo* 15 medium for 45 min. Cells were washed and stimulated with 2,000 IU/ml of recombinant IFN-α (PBL) for 12 min.

Mouse spleens went through RBC lysis and made into single cell suspensions. CD4^+^ T cells were positively isolated (Miltenyi Biotec) and placed into wells that were coated with anti-CD3/CD28 (5 μg/ml; 145-2c11/37.51; BioXcell and UCSF Monoclonal Antibody Core). Murine cells were cultured in complete media containing RMPI-1640 supplemented with 10% FBS, 1% non-essential amino acids, 1% sodium pyruvate, 1% GlutaMAX, and 0.1% β-ME. For IL-12 stimulation, cells were activated for 72 h and let rest for 24 h. Cells were then stimulated with IL-12 for 20 min and subsequently analyzed for intracellular pSTAT3. For Th1 skewing assays, cell cultures were supplemented with the following Th0 and Th1 cytokines respectively; anti-IFN-γ (30 μg/ml; BioXcell) and anti-IL-4 (20 μg/ml; BioXcell); IL-2 (50 ng/ml; Peprotech), IL-12 (20 ng/ml; R&D systems), and anti-IL-4 (20 μg/ml; BioXcell). At 48 h, cells were split into two wells and fresh media was added to each condition with the respective cytokines described above. Cells were harvested on day 5 and examined for intracellular IFN-γ. For IL-23/pSTAT3 stimulation, total splenocytes were cultured in media containing anti-CD3 (2.5 μg/ml; BioXcell), IL-6 (30 ng/ml), TGF-β (3 ng/ml; R&D systems), and anti-IFN-γ (10 μg/ml). At 72 h, anti-IFN-γ and IL-23 (10 ng/ml; R&D systems) were added to the cultures. Cells were harvested on day 6 and stimulated with IL-23 for 15 min at 37°C. CD4^+^ T cells were than analyzed for intracellular pSTAT3. For Th17 skewing assays, total splenocytes were cultured with the same supplements as described for IL-23/pSTAT3 stimulation above. At 72 h, anti-IFN-γ and IL-23 were added to the cultures on day 3, 7, and 10. On day 12, cells were stimulated with PMA (50 ng/ml; EMD Millipore), Ionomycin (1 μg/ml; Sigma-Aldrich), and Monensin (20 ng/ml; eBioscience) to be analyzed for intracellular IL-17.

### *In vitro* Tfh Generation

Splenic CD4^+^ T cells were isolated as described above. All cells were stimulated with anti-CD3/CD28 coated beads (Thermo Fisher), with IL-2 (Peprotech) and supplemented with the following for Th0 and Tfh, respectively: anti-IFN-γ (30 μg/ml; BioXcell) and anti-IL-4 (20 μg/ml; BioXcell); IL-12 alone (20 ng/ml). Beads were removed after 48 h of stimulation and fresh media was added to each condition with the respective cytokines described above. Cells were harvested six days after initial stimulation to assess for Tfh surface markers.

### *In vitro* GC Stimulation

Splenic B cells were purified from mice with CD43^+^ depletion (Miltenyi Biotec). Cells were cultured in complete media (RMPI-1640 supplemented with 10% FBS, 1% penicillin-streptomycin, 1% sodium pyruvate, 1% Hepes, 1% GlutaMAX, and 0.1% β-ME) for 48 h at 37°C. B cells were stimulated with or without the following reagents; R848 (5 ng/ml); anti-mouse IgM F(ab')_2_ fragment (1 μg/ml; Jackson ImmunoResearch, Inc.); anti-mouse CD40 (1 μg/ml; SouthernBiotech); IL-12 (20 ng/ml). Supernatant was collected and evaluated with an IL-6 ELISA (eBioscience).

### *In vivo* Immunizations

VLPs were made with bacteriophage Qβ capsid protein that contain single-stranded RNA which were kindly provided by Dr. Baidong Hou (39). Mice were injected with 2 μg of VLPs i.p. Twelve days post-immunization, spleens and serum were harvested from the mice. Cells were stained with surface markers for Tfh and GC B cells. Serum was analyzed for VLP-specific antibodies as previously described (40).

Mice were immunized by i.p. with 200 μl of PBS containing 20% sheep red blood cells (SRBCs). Spleens and serum were harvested on day 5 to assess surface markers and total IgG1 by ELISA.

### Bone Marrow Transplantation

BM was harvested from the femora and tibiae of *Was*^−/−^*.Tyk2*^*P*^, *Was*^−/−^*.Tyk2*^*NP*^*, Was*^−/−^*.Tyk2*^*NP*/*P*^ and *Was*^−/−^*.IL12R*^−/−^ mice. Single cell suspensions were depleted for CD138^+^ cells (Miltenyi Biotec). CD138-depleted *Was*^−/−^*.Tyk2*^*P*^, *Was*^−/−^*.Tyk2*^*NP*^, *Was*^−/−^*.Tyk2*^*NP*/*P*^ and *Was*^−/−^*.IL12R*β*2*^−/−^ donor BM was mixed with respective *Tyk*2^*P*^.μ*MT*, *Tyk*2^*NP*^.μ*MT*, *Tyk*2^*NP*/*P*^.μ*MT*, and *IL12R*β*2*^−/−^ μ*MT* at a 20:80 ratio, and 6 × 10^6^ total BM cells were injected retro-orbitally into lethally irradiated mice (450cGy x 2 doses) to generate WAS chimeras in which all hematopoietic lineages express the variant *Tyk*2^*P*^allele. Resulting BM chimeras were bled at 12 weeks and 24 weeks post-transplant date by retro-orbital puncture and sacrificed at 24–26 weeks post-transplant. Serum dsDNA antibodies were assessed as previously described (37).

### Experimental Autoimmune Encephalomyelitis (EAE)

EAE was induced with s.c. immunization of the flanks with an emulsified mixture containing CFA, MOG_35−55_ peptide (100 μg), and Mycobacterium tuberculosis extract H37Ra (4 μg/ml; Difco). Each animal also received i.p. immunization of pertussis toxin (200 ng) on days 0 and 2. Mice were assessed daily for clinical symptoms of EAE and scored according to the following criteria: 0-no signs of disease; 1-limp tail; 2-hind limb weakness; 3-hind limb paralysis; 4-hind limb, and forelimb paralysis.

### Flow Cytometry

PBMCs were thawed, washed with PBS and rested in *X-vivo* 15 medium (Lonza) at 37°C and 5% CO_2_ for 45 min. Cells were washed with PBS and 1 × 10^6^ cells were stained in FACS buffer (PBS/0.5% BSA/0.1 NaN_3_) with a cocktail of fluorophore-conjugated antibodies at RT for 20 min. For human IL-12/pSTAT signaling, cells were fixed and permeabilized using Fix buffer I (BD Biosciences) and Perm buffer III (BD Biosciences), respectively, according to the manufacturer's instructions. Cells were washed and stained simultaneously for surface markers and intracellular pSTAT3 and pSTAT4 at RT for 45 min. For human IFN-α, cells were washed and stained simultaneously for surface markers and intracellular pSTAT1 at RT for 45 min. IFNAR surface levels were determined in unfixed, non-permeabilized cells. The following antibodies were used for the detection of proteins in human samples: CD3-AF700 (UCHT1), CXCR3-PE (G025H7), CCR6-PerCP/Cy5.5 (G034E3), PD1-BV605 (EH12.2H7), CD3-PerCp/Cy5.5 (UCHT1), CD19-AF700 (HIB19), IgM-FITC (MHM-88), CD38-PE-Cy5 (Hit5), CD24-BV510 (ML5), CD10-PE-Cy7 (HI10a), CD8-PerCP/Cy5.5 (RPA-T8), CD45RA-PE-Cy7 (HI100), CD56-BV421 (HCD56), from BioLegend; CD8-PE-Cy7 (SFCI21Thy2D3) from Beckman Coulter; CD4-BV510 (SK3), CD45RA-PE-Cy5 (HI100), CXCR5-BV421 (RF8B2), CD27-BV605 (L128), IL-12Rb1- APC (2.4E6), pSTAT3-PE (4/P-STAT3), pSTAT4-PerCP/Cy5.5 (38/p-Stat4), pSTAT1-AF488 (4a) from BD Biosciences; IFNAR-APC (85228) from R&D Systems. The following antibodies were used for the detection in mouse samples: pSTAT3-Alexa647 (4/P-STAT3), CXCR5-Biotin (2G8), Streptavidin-PE-Cy7, CD19-PE-Cy7 (1D3), FAS-PE (Jo2) from BD Biosciences; CD4-Pacific Blue (RM4-5), IFNg-APC (XMG1.2), B220-PerCP-Cy5.5 (RA3-6B2), IL-17A-PE (TC11-18H10.1), IL-17A-PerCP-Cy5.5 (TC11-18H10.1) were from BioLegend; CD3-APC-eFLuor780 (17A2), GL7-Alexa 647 (GL-7), PE-IFNg (XMG1.2), PE-Cy5 (GK1.5), CD3e-Fitc (eBio500A2), CD3e-PerCP-Cy5.5 (145-2c11) were from eBioscience; CD4-APC (GK.1) were from Southern Biotech; PD1 Fitc (J43) were from Life Technologies; PNA-FITC (FI-1071) were from Vector Labs. Live/Dead Near-IR Dead Cell Stain Kit (Invitrogen) was used to assess viability by flow cytometry. Alexa Fluor 647-labeld Q-VLP was kindly provided by Dr. Baidong Hou (39, 41). FlowJo (version 10) was used for data analysis.

### Statistical Analysis

All statistical analysis was performed using GraphPad Prism (version 7). All statistical tests and *P*-values are specified in the figure legends.

## Results

### Healthy Subjects With the *TYK2*^*P*^ Variant Exhibit a Decrease in Both Tfh Cells and Switched Memory B Cells

**Figure 1:**
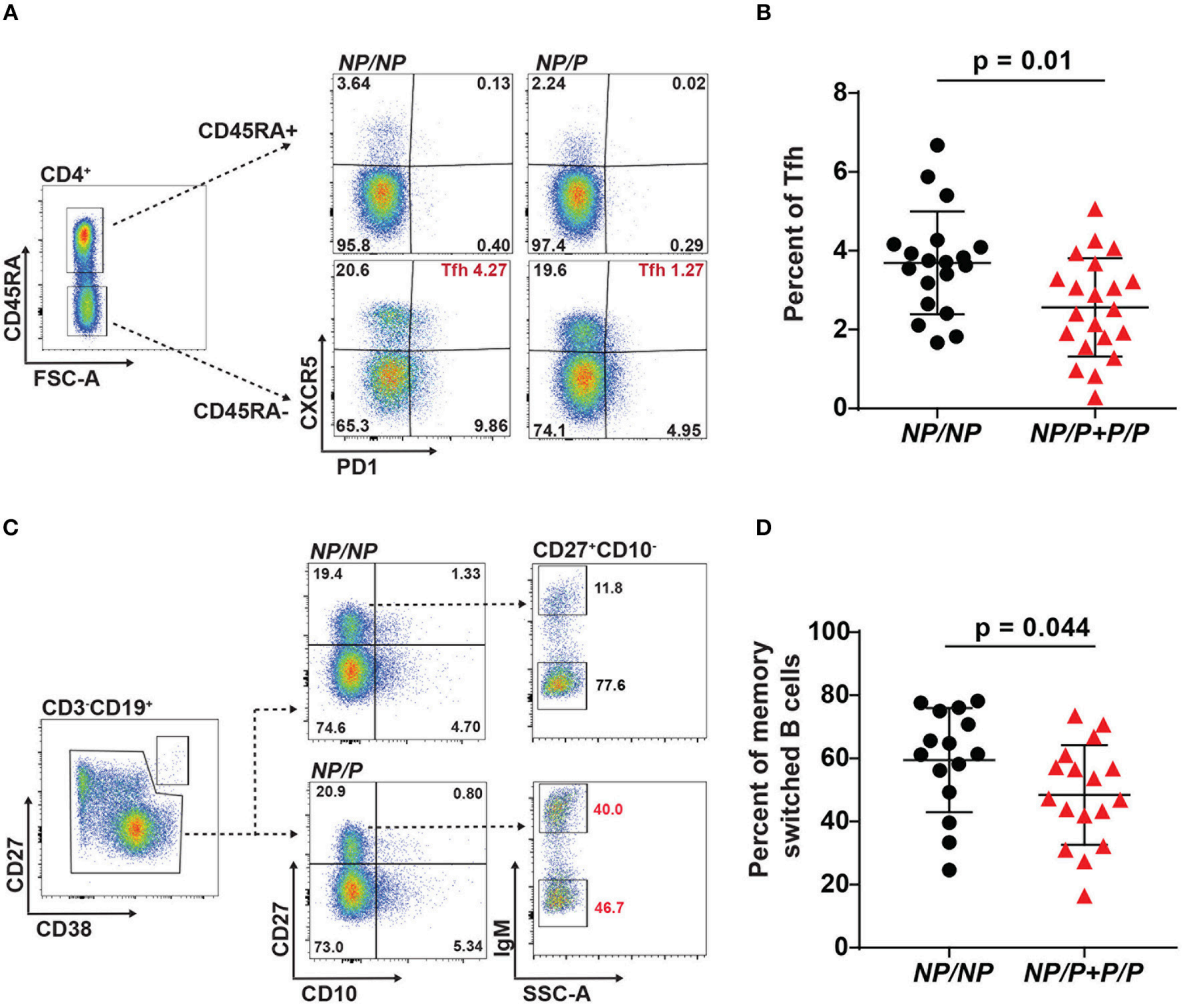
Healthy subjects expressing the *TyK*2 protective variant exhibit decreased proportion of circulating Tfh and switched memory B cells. **(A)** Gating strategy for T follicular helper (Tfh) cells, defined as CD4^+^CD45RA^−^CXCR5^+^PD1^+^ T cells. Shown are representative dot plots of the Tfh cell frequency in subjects with *TYK2*^*NP*/*NP*^ (*NP/NP*), *TYK2*^*NP*/*P*^ (*NP/P*), or *TYK2*^*P*/*P*^ (*P/P*) (non-protective (*NP*) vs. protective (*P*) alleles of rs34536443; encoding for Alanine-1104 vs. Proline-1104). **(B)** Quantification of Tfh cell frequency. **(C)** Gating strategy for switched memory B cells, defined as plasmablast (PB) negative CD3^−^CD19^+^CD27^+^CD10^−^IgM^−^ B cells. Shown are representative dot plots of the switched memory B cell frequency in subjects. **(D)** Quantification of switched memory B cell frequency (Each symbol represents an individual donor) **(B,D)**; small horizontal lines indicate the mean (± s.d.). Data from a combined total of *n* = 19 *Tyk2*^*NP*/*NP*^ donors, *n* = 19 *TYK2*^*NP*/*P*^ donors, and *n* = 2 *TYK2*^*P*/*P*^ donors **(A,B)**; *n* = 15 *TYK2*^*NP*/*NP*^ donors, *n* = 13 *TYK2*^*NP*/*P*^ donors, and *n* = 4 *TYK2*^*P*/*P*^ donors **(C,D)**. Statistical analysis indicated from a Mann-Whitney U **(B,D)**.

To evaluate the effect of *TYK2*^*P*^ on lymphocyte populations, we examined peripheral blood mononuclear cells (PBMC) in healthy individuals with no family history of autoimmunity. Specifically, we assessed adaptive immune cells which require TYK2-dependent pathways for development and activation (27). Thawed PBMCs were stained with fluorophore-conjugated antibodies for a panel of T and B cell subset markers and analyzed by flow cytometry. We observed no effect of *TYK2*^*P*^ on the frequency of CD4^+^ naïve (RA^+^) and memory (RA^−^) T cells or of total CD3^−^CD19^+^ or memory CD3^−^CD19^+^CD27^+^CD10^−^ B cells. In contrast, we found that individuals expressing the *TYK2*^*P*^ allele have decreased circulating CD4^+^CD45RA^−^PD1^+^CXCR5^+^ Tfh cells (Figures 1A,B). Consistent with the role for Tfh cells in promoting B cell GC responses, we also observed a reduced frequency of CD3^−^CD19^+^CD27^+^CD10-IgM^−^ switched memory B cells (Figures 1C,D) in individuals with the protective allele. Thus, individuals expressing *TYK2*^*P*^ exhibited low frequencies of Tfh cells, essential for germinal center formation, and switched memory B cells, products of germinal centers, suggesting that TYK2 plays a role in cytokine pathways important for regulation of germinal centers and immune activation.

### *In vitro* IL-12 Driven Tfh Generation and B Cell IL-6 Production Is Decreased Using Murine *Tyk2*^*P*^ Cells

To gain better understanding of the function of TYK2^P^ in cytokine signaling and autoimmune disease, we generated a knock-in mouse strain containing the identical amino-acid substitution in the murine TYK2 protein (*Tyk2-P1124A*), hereafter referred to as *Tyk2*^*P*^ mice or as *Tyk2*^*NP*/*P*^ and *Tyk2*^*P*/*P*^ for heterozygous and homozygous animals, respectively (further detailed explanation of genotypes, please see Supplementary Table 1). To generate founder mice, we used homologous recombination on the non-autoimmune prone C57BL/6 genetic background (Supplementary Figures 1A–C). Gene targeting produced the variant coding change (encoding the substitution P1124A) in exon 21 of *Tyk2* (Supplementary Figure 1B). Based upon our targeting strategy, we also crossed *Tyk2*^*P*^ mice with a murine line ubiquitously expressing CRE to create *Tyk2* knockout (*Tyk2*^−/−^) animals of an identical genetic background for use in some studies (Supplementary Figures 1A,C).

**Figure 2:**
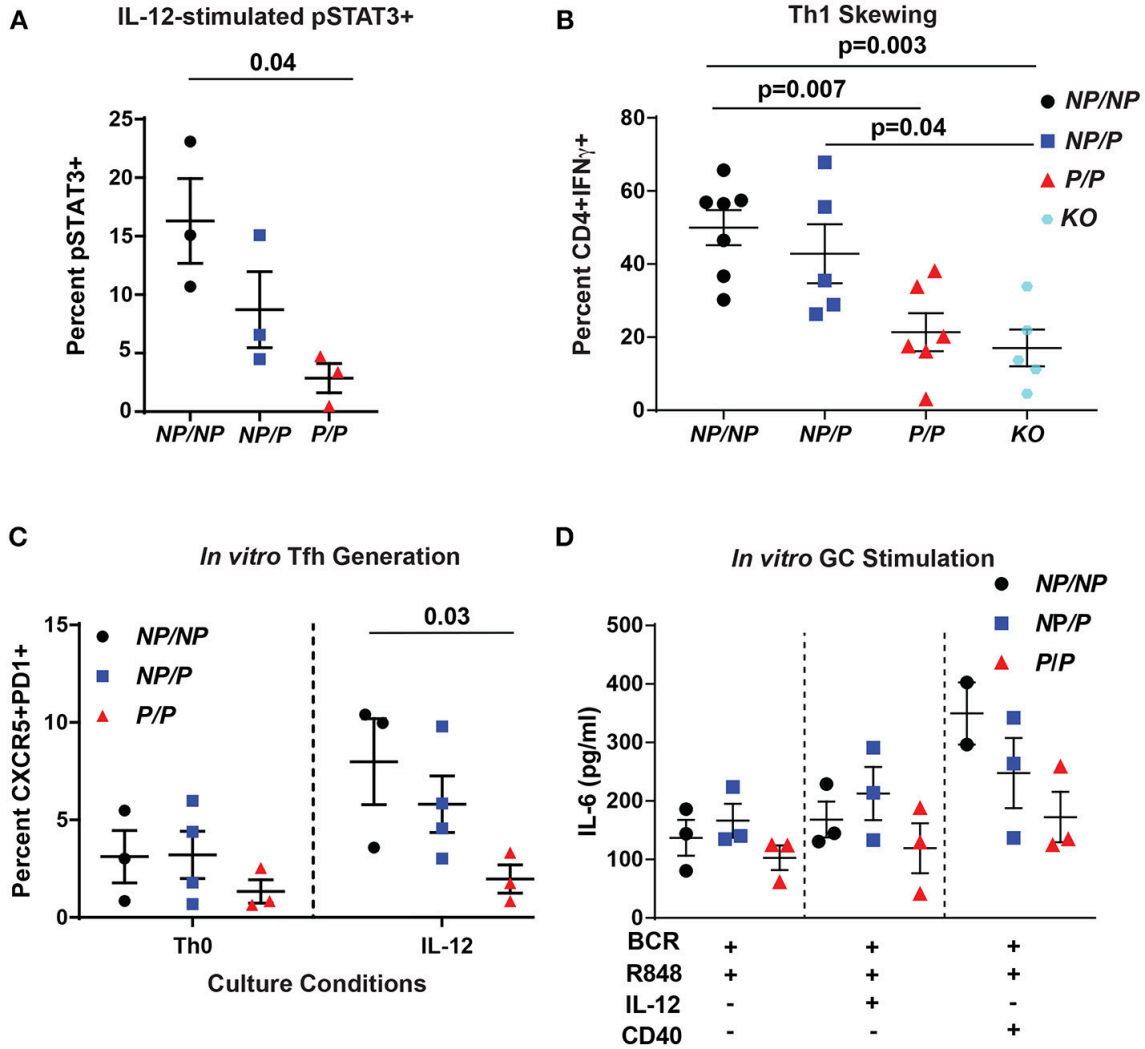
Murine *Tyk2*^*P*^ lymphocytes exhibit decreased IL-12 signaling and reduced *in vitro* generation Tfh cells and IL-6 production in response to GC programming. **(A–C)** Splenic CD4^+^ T cells were isolated from *Tyk2*^*NP*/*NP*^ (*NP/NP*), *Tyk2*^*NP*/*P*^ (*NP/P*), *Tyk2*^*P*/*P*^ (*P/P*), or *Tyk2*^−/−^ *(KO*) mice. **(A)** CD4^+^ T cells were stimulated with 2 ng/mL of IL-12 for 20 min and assessed for phosphorylation of STAT3 (pSTAT3) using flow cytometry. **(B)** Frequency of IFN-γ^+^ CD4^+^ T cells following *in vitro* Th1 skewing culture conditions with IL-12, IL-2, and anti-IL-4 for five days and analyzed by flow cytometry. **(C)** Frequency of Tfh-like T cells (CD4^+^ CXCR5^+^PD1^+^) following culture with the indicated cytokines for six days. **(D)** Splenic B cells were isolated from *Tyk2*^*NP*/*NP*^ (*NP/NP*), *Tyk2*^*NP*/*P*^ (*NP/P*), or *Tyk2*^*P*/*P*^ (*P/P*) mice. IL-6 production from B cells stimulated with the indicated cytokines for 48 h. Small horizontal lines indicate the mean (± s.e.m.). Statistical analysis were performed using Friedman Test with Dunn's multiple comparison **(A)**, one-way ANOVA with Tukey's multiple comparisons test **(B,D)**, and two-way ANOVA with Tukey's multiple comparisons test **(C)**. Data are derived from three **(A,C,D)** or seven independent experiments **(B)**. Each symbol represents an individual biological replicate (individual mouse); *Tyk2*^*NP*/*NP*^ *n* = 3, *Tyk2*^*NP*/*P*^ *n* = 3, or *Tyk2*^*P*/*P*^ *n* = 3 **(A,D)**; T*yk2*^*NP*/*NP*^ *n* = 7, *Tyk2*^*NP*/*P*^ *n* = 5,*Tyk2*^*P*/*P*^ *n* = 6 or *Tyk2*^*KO*^ *n* = 5 **(B)**; *Tyk2*^*NP*/*NP*^ *n* = 3, *Tyk2*^*NP*/*P*^ *n* = 4, or *Tyk2*^*P*/*P*^ *n* = 3 **(C)**.

The TYK2-dependent IL-12 cytokine pathway is important for Tfh generation by promoting phosphorylation of STAT3 (pSTAT3) (12). To test pSTAT3 levels in murine cells, we isolated CD4^+^ T cells from littermate control (*Tyk2*^*NP*/*NP*^), heterozygous *(Tyk2*^*NP*/*P*^) or homozygous (*Tyk2*^*P*/*P*^) and assessed for IL-12-induced pSTAT3. We found that homozygous *Tyk2*^*P*/*P*^ cells exhibited diminished pSTAT3 (Figure 2A). Further, *Tyk2*^*P*/*P*^ CD4^+^ T cells were also unable to skew toward a Th1 phenotype *in vitro*, a process also dependent on IL-12 signaling (Figure 2B). Similar to previously published findings, *Tyk2*^−/−^ CD4^+^ T cells exhibited a similar decrease in the capacity to skew toward a Th1 phenotype (24, 42). These data mirrored our findings using *Tyk2*^*P*/*P*^ CD4^+^ T cells implying that the protective allele encoded for a TYK2 protein with reduced functional activity. Despite the decreased Tfh cells in the circulation in human subjects heterozygous for the protective variant, we could not discern significant differences in IL-12-induced pSTAT3 or pSTAT4 using primary human CD4^+^ T cells from a cohort of heterozygous healthy subjects (Supplementary Figures 2A–H).

To explore the role of TYK2^P^ in IL-12-induced Tfh cell generation, we used an *in vitro* assay to examine this question. *Tyk2*^*P*/*P*^ CD4^+^ T cells were not able to generate Tfh-like cells as efficiently as *Tyk2*^*NP*/*NP*^ cells in response to IL-12 alone (Figure 2C). Based on the diminished switched memory population in healthy donors with the protective variant (Figure 1D), we assessed the role of IL-12 signaling in modulating the activation of *Tyk2*^P^ murine B cells. Previous work has implicated IL-12 in promoting B cell activation and antibody production (43, 44). We used an *in vitro* “GC-like” stimulation with and without the addition of IL-12 and monitored the production of IL-6. IL-6 is produced by activated B cells and promotes GC B and Tfh cell development (45), and B cell intrinsic IL-6 production is required for autoimmune GC B cell responses (46). Under all conditions, *Tyk2*^*P*/*P*^ B cells exhibited a trend for diminished IL-6 production compared to control *Tyk2*^*NP*/*NP*^ or heterozygous *(Tyk2*^*NP*/*P*^) B cells (Figure 2D) but these differences did not reach statistical significance. In summary, diminished *in vitro* Tfh-like and Th1 generated T cells from *Tyk2*^*P*/*P*^ mice were most likely secondary to diminished IL-12 signaling.

### *Tyk2*^*P*^ Does Not Impact Tfh and GC B Cell Formation Following T-Dependent Immunization

**Figure 3:**
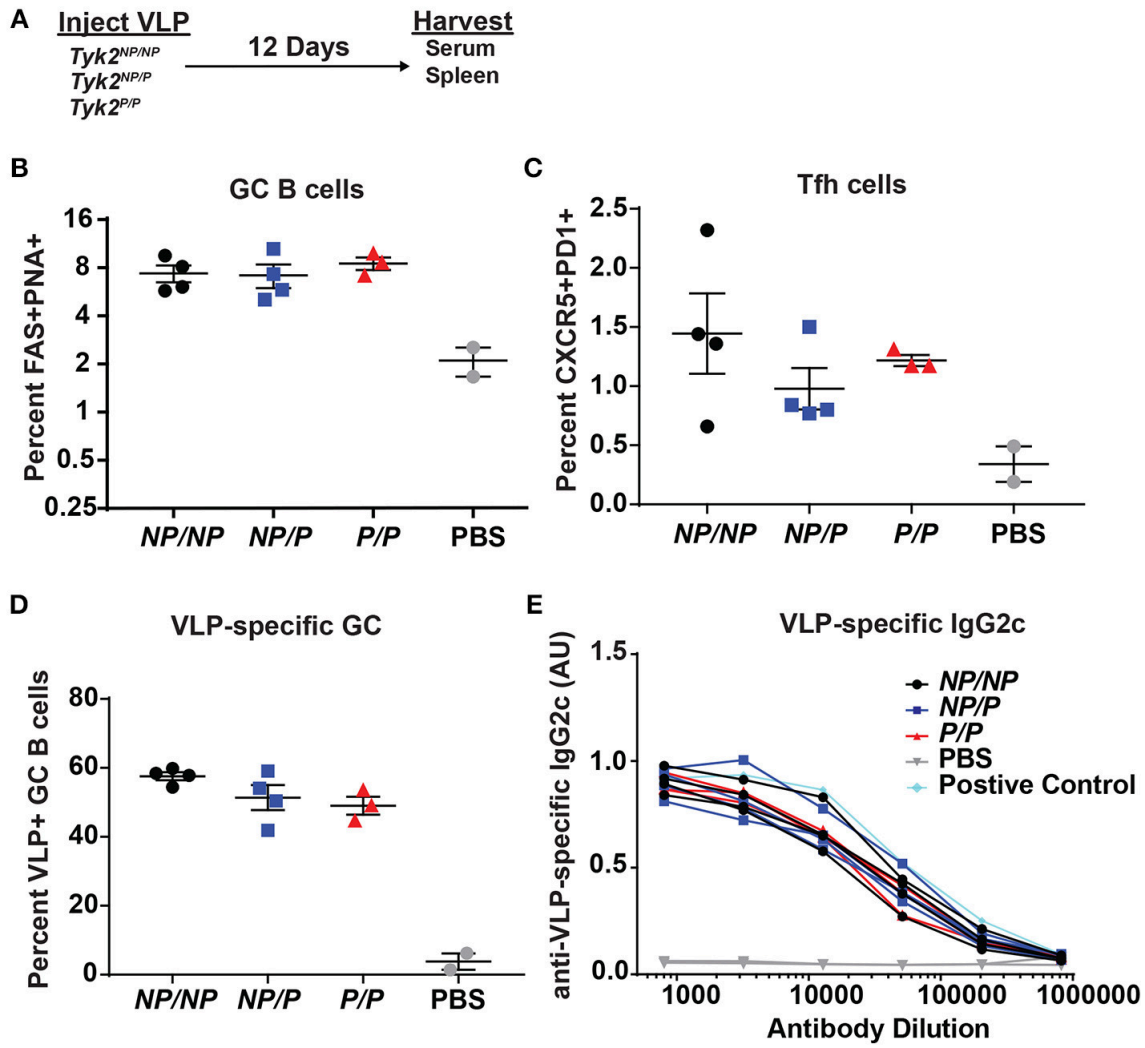
Tfh and GC B cell formation *in vivo* in response to immunization with virus-like particles is not impacted by *Tyk2*^*P*^ expression. **(A)** Experimental schematic for VLP immunization. *Tyk2*^*NP*/*NP*^ (*NP/NP*), *Tyk2*^*NP*/*P*^ (*NP/P*), or *Tyk2*^*P*/*P*^ (*P/P*) mice were immunized i.p. with 2 μg of TLR7-loaded virus-like particles (VLP) or with PBS as control. Splenocytes were analyzed for frequency of: **(B)** germinal center (GC) B cells (B220^+^FAS^+^PNA^+^); **(C)** Tfh cells (CD4^+^CXCR5^+^PD1^+^); and **(D)** VLP+ GC B cells at day 12 post-immunization. **(E)** Sera was collected at day 12 post-immunization and assessed by ELISA for VLP-specific IgG2c antibodies. Small horizontal lines indicate the mean (± s.e.m.). ELISA results are displayed as absorbance at 450 nm normalized to results using a blank well and presented in arbitrary units (AU) **(E)**. Representative data are shown from one of two independent experiments **(B–E)**. Statistical analysis was performed using one-way ANOVA with Tukey's multiple comparisons test **(B–D)**. Each symbol represents an individual biological replicate; *Tyk2*^*NP*/*NP*^ *n* = 4, *Tyk2*^*NP*/*P*^ *n* = 4, *Tyk2*^*P*/*P*^ *n* = 3, PBS *n* = 2, or Positive Control *n* = 1 **(B–E)**.

Next, based on its impact on Tfh cells *in vitro* and in human subjects, we examined the effect of *Tyk2*^*P*^ on generation of Tfh and GC B cells *in vivo*. We first assessed T cell-dependent immunization using TLR7-loaded virus-like particles (VLP) in control (*Tyk2*^*NP*/*NP*^) mice, mice heterozygous *(Tyk2*^*NP*/*P*^), or homozygous (*Tyk2*^*P*/*P*^) for the protective variant. At the peak of the immune response, there was no difference in the proportion or number of Tfh cells or GC B cells generated by these strains (Figures 3A–C). Additionally, we saw no differences in VLP-specific GC B cells or in high-affinity anti-VLP IgG2c antibodies (Figures 3D,E). We expanded upon this result by using a second immunization strategy designed to promote a more sustained GC response triggered via delivery of sheep red blood cells (SRBCs) and also included cohorts of *Tyk2*^−/−^ animals. Again, all strains exhibited equivalent production of GC B cells, Tfh cells, and antibodies (Supplemental Figures 3A–D). Taken together, *Tyk2*^P^ appears to have little or no impact on T-dependent GC and antibody formation in response to immunization strategies that rely on the formation and function of Th1/Th2 cells.

### *Tyk2^*P*^* Does Not Affect Tfh and GC B Cell Formation in Murine Lupus Models

**Figure 4:**
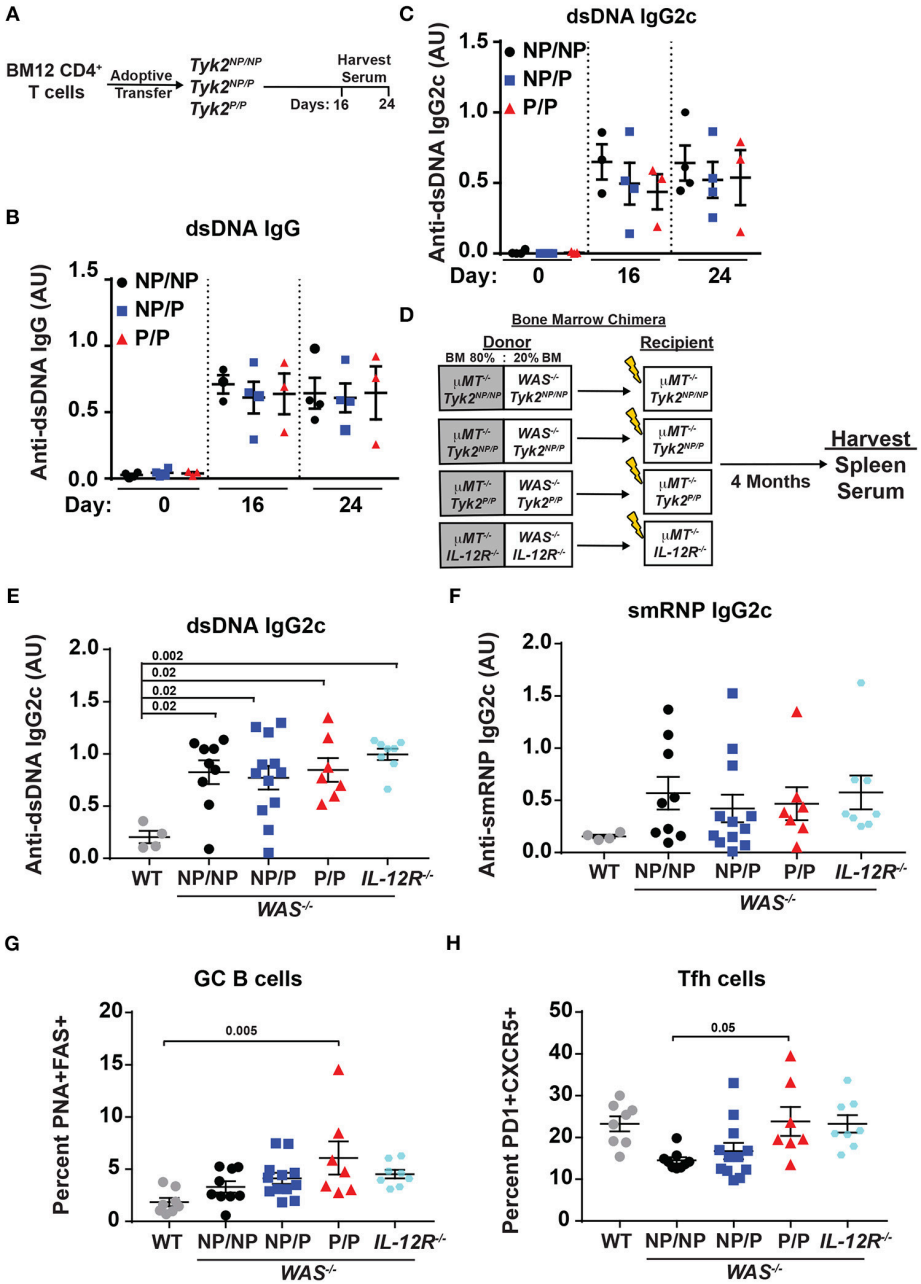
*Tyk2*^*P*^ and *IL*−*12R*^−/−^ mice are not protected in murine models of lupus. **(A)** Experimental schematic for BM12 CD4^+^ T cell adoptive transfer model. 5.0 × 10^6^ BM12 CD4^+^ T cells were adoptively transferred into *Tyk2*^*NP*/*NP*^, *Tyk2*^*NP*/*P*^, or *Tyk2*^*P*/*P*^ recipient mice and autoantibodies and splenic cell populations (not shown) were assessed at indicated times. Serum ELISA analysis for: **(B)** anti-dsDNA IgG and **(C)** anti-dsDNA IgG2c autoantibodies. **(D)** Schematic for establishment of B cell-specific bone marrow (BM) chimeras using an 80%:20% mixture of bone marrow from μ*MT*^−/−^ mice and WT or Wiskott-Aldrich knock-out (*WAS*^−/−^) donor cells with the indicated *Tyk2* or *IL-12R* alleles (*WAS*^−/−^*Tyk2*^*NP*/*NP*^*, WAS*^−/−^*Tyk2*^*NP*/*P*^*, WAS*^−/−^*Tyk2*^*P*/*P*^*, WAS*^−/−^*IL-12R*^−/−^), respectively. Yellow lightning bolt represents irradiation of recipient mice. See methods for additional details of experimental design. **(E,F)** ELISA analysis of serum at 16 week post-transplantation for: **(E)** anti-dsDNA IgG2c and **(F)** anti-smRNP IgG2c autoantibodies in indicated recipient mice. **(G,H)** Splenocytes were isolated at 16 week and analyzed by flow cytometry for frequency of: **(G)** GC and **(H)** Tfh cells as described in Figure 2. Small horizontal lines indicate the mean (± s.e.m.). ELISA results are displayed as absorbance at 450 nm normalized to results of a blank well and presented in arbitrary units (AU). **(B,C,E,F)** Statistical analysis was performed using one-way ANOVA with Tukey's multiple comparisons test. Data are representative of two independent experiments **(B,C)** or data combined from two independent experiments **(E–H)**. Each symbol represents an individual biological replicate;*Tyk2*^*NP*/*NP*^ *n* = 3, *Tyk2*^*NP*/*P*^ *n* = 4,*Tyk2*^*P*/*P*^ *n* = 3 or PBS *n* = 2 **(A-C)**; WT *n* = 4,*Tyk2*^*NP*/*NP*^ *n* = 9, *Tyk2*^*NP*/*P*^ *n* = 12,*Tyk2*^*P*/*P*^ *n* = 7 or *IL-12R*^−/−^ *n* = 8 **(E,F)**; WT *n* = 8,*Tyk2*^*NP*/*NP*^ *n* = 9, *Tyk2*^*NP*/*P*^ *n* = 12,*Tyk2*^*P*/*P*^ *n* = 7 or *IL-12R*^−/−^ *n* = 8 **(G,H)**.

*TYK2*^*P*^ has been associated with protection from multiple autoimmune diseases including SLE (32). Therefore, we next directly assessed the role of the protective variant in disease development using alternative murine lupus models utilizing *Tyk2*^*P*^ mice. As an initial test, we used the BM12 T cell adoptive transfer model of lupus. The BM12 strain was derived from C57BL/6 mice and contains a three-amino-acid change in the major histocompatibility complex class II molecule H2-AB1^b^ (47). An autoimmune GC response is generated when BM12 CD4^+^ T cells are adoptively transferred into C57BL/6 recipients leading to production of autoantibodies directed against dsDNA within ~3 weeks following the cell transfer (48, 49). Therefore, to assess the impact of *Tyk2*^*P*^ in this setting, we transferred BM12 CD4^+^ T cells into control (*Tyk2*^*NP*/*NP*^), heterozygous *(Tyk2*^*NP*/*P*^), or homozygous (*Tyk2*^*P*/*P*^) recipient mice. Following CD4^+^ T cell transfer, there was no difference in the proportion of Tfh and GC B cells in any strain (data not shown). We also observed no differences in autoantibody levels at disease peak (Figures 4A–C).

To further examine the role of *Tyk2*^*P*^ in murine lupus, we utilized the Wiskott-Aldrich Deficient (*WASp*^−/−^) B cell bone marrow (BM) chimera lupus model (38, 46, 50–53). In chimeras with B cell intrinsic loss of *WASp*^−/−^, mice display spontaneous GCs, autoantibodies, renal histopathology, and early mortality (38). In order to assess the impact of various *Tyk2*^*P*^ alleles in all relevant cell lineages in the development of lupus in this model, we first intercrossed μ*MT*^−/−^ mice with our knock-in strain to establish μ*MT*^−/−^ mice co-expressing *Tyk2*^*NP*/*NP*^, *Tyk2*^*NP*/*P*^, or *Tyk2*^*P*/*P*^, respectively. As shown schematically in Figure 4D, cohorts of animals for each of these μ*MT*^−/−^ strains were lethally irradiated and reconstituted by BM transplantation using a mixture of 80% μ*MT*^−/−^ BM (expressing *Tyk2*^*NP*/*NP*^, *Tyk2*^*NP*/*P*^, or *Tyk2*^*P*/*P*^, respectively) and 20% *WAS*^−/−^ BM (co-expressing *Tyk2*^*NP*/*NP*^, *Tyk2*^*NP*/*P*^, or *Tyk2*^*P*/*P*^, respectively; Figure 4D). As an additional control to assess the impact of IL-12 receptor signaling in disease development, we utilized μ*MT*^−/−^ recipient strains and donor BM cells both deficient for IL-12Rβ2 (Figure 4D). Strikingly, all recipients of *WAS*^−/−^ BM developed high-titer class-switched IgG2c anti-dsDNA and anti-smRNP antibodies within 4 months post-transplant. We also observed no differences in relative levels of autoantibody production, GC B cells, or Tfh cells between recipients with alternative *Tyk2*^*P*^ alleles (Figures 4D–H). Moreover, despite the anticipated role for IL-12 in modulating T and B cell activation, IL-12Rβ2 deficiency exerted no appreciable impact on disease within this model with recipients developing autoantibodies, GC B cells, and Tfh cells as efficiently as *WAS*^−/−^ chimeras (Figures 4D–H). Taken together, these findings suggest that *Tyk2*^*P*^ does not play a major role in development of autoimmune GC responses or in modulating autoantibody production in murine SLE.

### Type I Interferon Signaling Is Reduced in T Cells From *TYK2*^P^ Healthy Subjects

**Figure 5:**
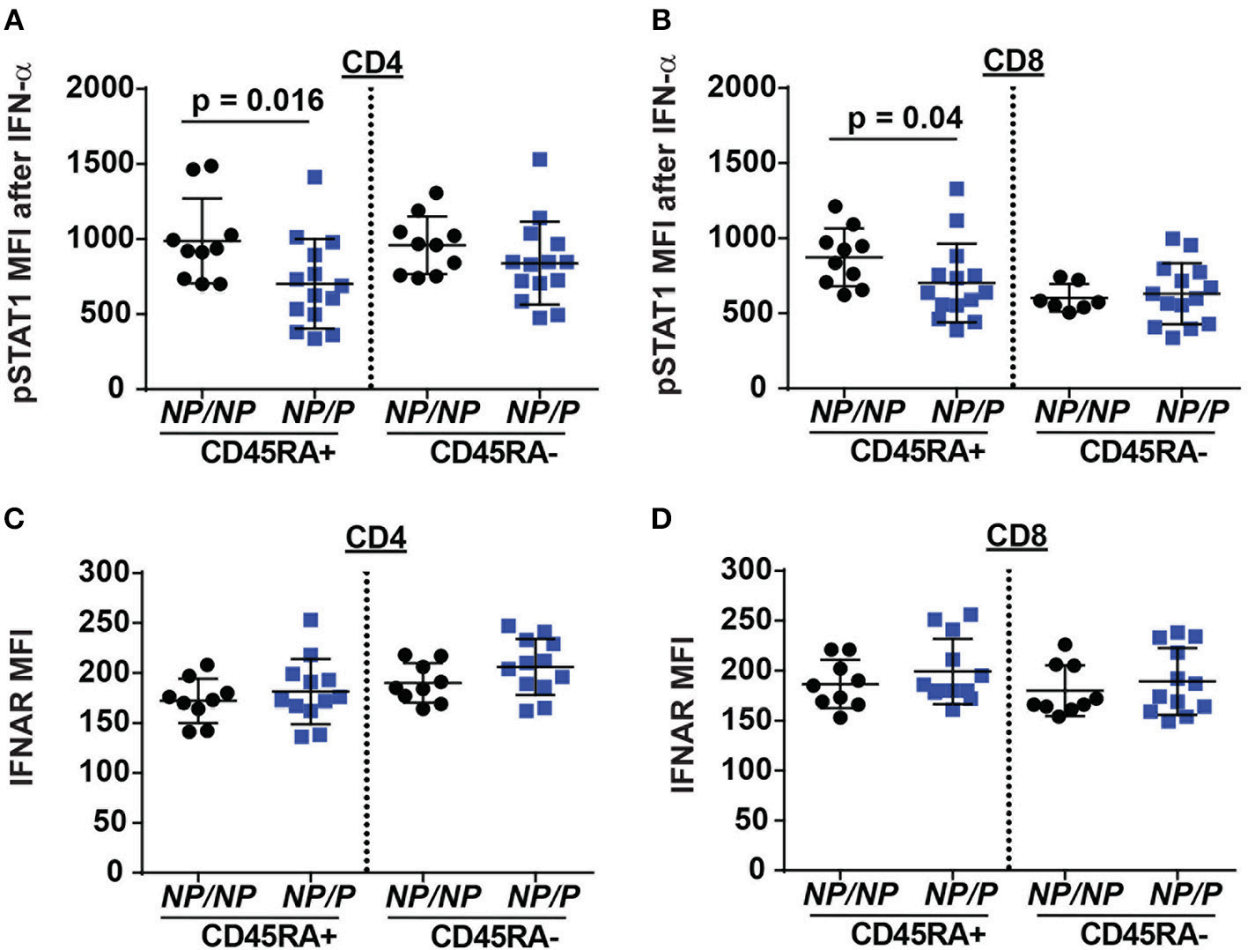
Decreased IFN-α/pSTAT1 signaling in naive T cells from healthy subjects expressing the TYK2 protective variant. PBMC from healthy subjects with *TYK2*^*NP*/*NP*^ (*NP/NP*) or heterozygote for the protective allele *TYK2*^*NP*/*P*^ (*NP/P*), were thawed and stimulated with 2,000 IU/ml recombinant IFN-α for 12 min and assessed using flow-cytometry for phosphorylation of STAT1 (pSTAT1) or IFN I receptor (IFNAR) surface expression. Quantification of pSTAT1 MFI following IFN-α stimulation in: **(A)** naive (RA+) and memory (RA-) CD4^+^ and **(B)** CD8^+^ T cells. Quantification of IFNAR mean fluorescence intensity (MFI) in: **(C)** naive (RA+) and memory (RA-) CD4^+^ and **(D)** CD8^+^ T cells. Each symbol represents an individual donor **(A–D)**; small horizontal lines indicate the mean (± s.d.). Data from a combined total of *n* = 10 *TYK2*^*NP*/*NP*^ donors or *n* = 14 *TYK2*^*NP*/*P*^ donors **(A–D)**. Statistical analysis performed using Mann-Whitney U testing **(A–D)**.

Another pathway with a requirement for TYK2 is type I interferon (IFN I) signaling (18). This pathway may also impact Tfh generation. To test the role of TYK2^P^ in type I interferon receptor (IFNAR) signaling, we stimulated PBMCs from healthy control subjects and subjects heterozygous for the protective variant using IFN-α and examined phosphorylated STAT1 (pSTAT1) levels following activation. Naïve *TYK2*^*NP*/*P*^ CD4^+^ and CD8^+^ T cells exhibited a decrease in IFN-α induced pSTAT1 levels compared to cells from control *TYK2*^*NP*/*NP*^ subjects (Figures 5A–D), a difference that was not due to altered IFNAR surface expression. These findings were consistent with a previous report showing diminished pSTAT1 and pSTAT3 levels following IFN-α stimulation in subjects with the protective allele (33). Thus, IFNAR signaling is reduced by the expression of TYK2^P^.

### *TYK2*^*P*^ Is Involved in IL-23 Signaling, Th17 Skewing, and Tfh-17 Formation

**Figure 6:**
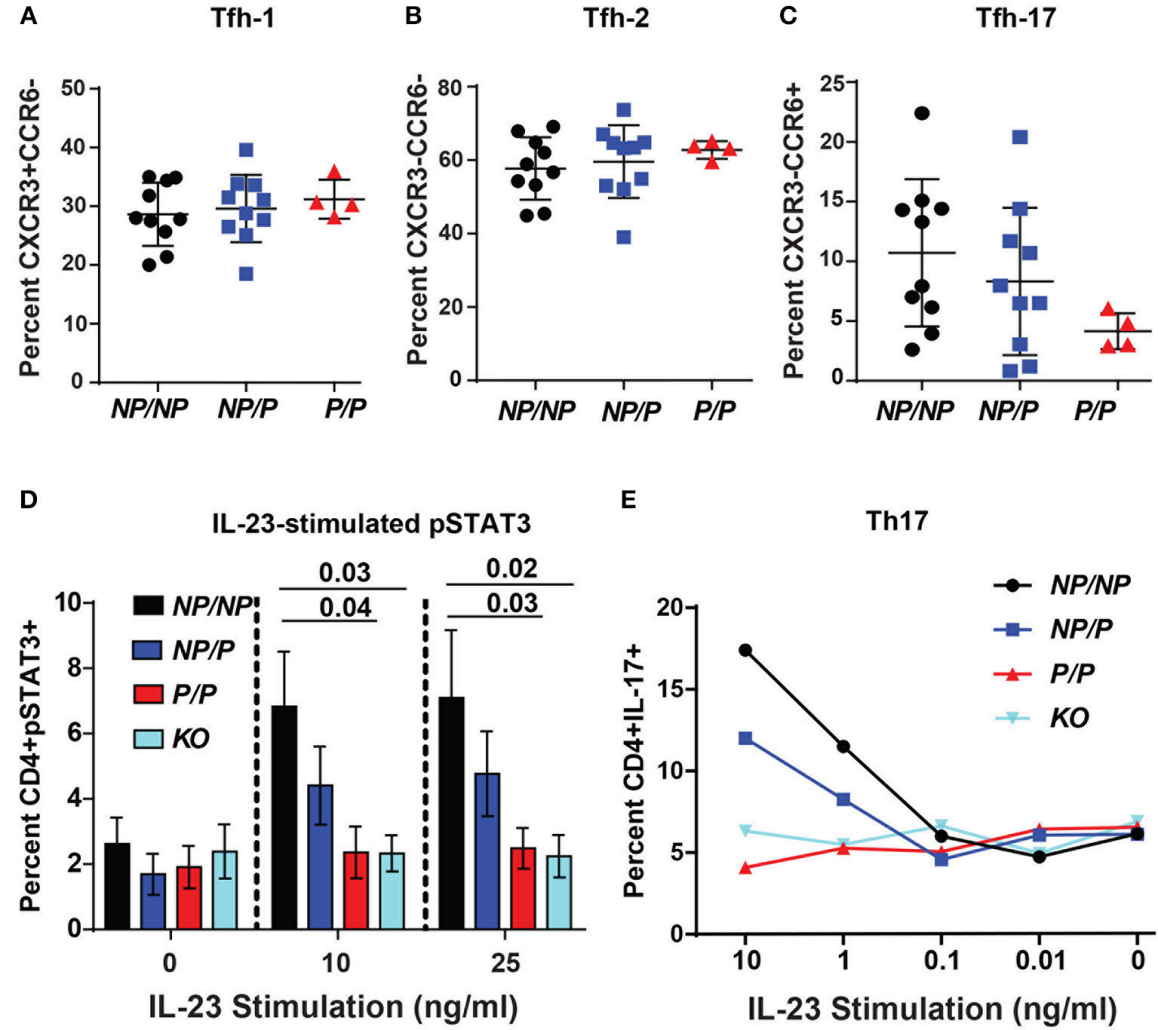
Healthy subjects expressing the TYK2 protective variant exhibit decreased circulating Tfh-17 cells and *Tyk2*^*P*^ mice exhibit reduced IL-23 signaling and Th17 skewing. **(A–C)**, Quantification of Tfh subsets in healthy subjects with *TYK2*^*NP*/*NP*^ (*NP/NP*), *TYK2*^*NP*/*P*^ (*NP/P*), or *TYK2*^*P*/*P*^ (*P/P*) alleles, respectively. Results of flow cytometry studies assessing the frequency of: **(A)**, CXCR3^+^CCR6^−^ Tfh-1; **(B)** CXCR3^−^CCR6^−^ Tfh-2; and **(C)** CXCR3^−^CCR6^+^ Tfh-17 T cells within the CD4^+^CXCR5^+^ cell population. Each symbol represents an individual donor. **(D,E)** Splenic CD4^+^ T cells were isolated from *Tyk2*^*NP*/*NP*^ (*NP/NP*), *Tyk2*^*NP*/*P*^ (*NP/P*), *Tyk2*^*P*/*P*^ (*P/P*) or *Tyk2*^−/−^ *(KO*) mice. **(D)** CD4^+^ T cells were stimulated with using the indicated amounts of IL-23 for 15 min and assessed using flow cytometry for phosphorylation of STAT3 (pSTAT3). **(E)** CD4^+^ T cells were cultured in Th-17 skewing conditions with indicated amounts of IL-23 and assessed for the frequency of IL-17^+^ CD4 (Th-17) T cells. **(A–C)** small horizontal lines indicate the mean (± s.d.) and **(D)** mean (± s.e.m.). Data from a combined total of *n* = 10 *TYK2*^*NP*/*NP*^ donors, *n* = 10 *TYK2*^*NP*/*P*^ donors, and *n* = 4 *TYK2*^*P*/*P*^ donors **(A–C)**. Data are representative of three independent experiments **(D)** and one experiment **(E)**. Statistical analysis were performed using Kruskal-Wallis **(A–C)** and two-way ANOVA with Tukey's multiple comparisons test **(D)**.

Circulating human Tfh cells are comprised of three distinct developmental subsets that can be discriminated based on relative surface expression levels of CXCR3, CCR6, and CCR7 (14). Therefore, we next investigated whether a specific Tfh lineage was preferentially impacted by expression of *TYK2*^*P*^. Though not statistically different, we discovered that individuals expressing the protective variant exhibited a trend for a decrease in the relative proportion Tfh-17 cells (*p* = 0.123) but exhibited no changes in the proportion of Tfh-1 or Tfh-2 cells (Figures 6A–C). Consistent with this data, TYK2 is activated downstream of the IL-23 receptor engagement (19). To further study the role of TYK2^P^ in Th17 commitment and in Tfh-17 cells, we investigated IL-23 signaling in the murine CD4^+^ T cells derived from control (*Tyk2*^*NP*/*NP*^), heterozygous *(Tyk2*^*NP*/*P*^) or homozygous (*Tyk2*^*P*/*P*^) mice and from *Tyk2*^−/−^ animals. Both *Tyk2*^*P*/*P*^ and *Tyk2*^−/−^ T cells exhibited decreased IL-23 dependent pSTAT3 and heterozygous *Tyk2*^*NP*/*P*^ T cells exhibited a trend consistent with an intermediate phenotype (Figure 6D). Further, both *Tyk2*^*P*/*P*^ and *Tyk2*^−/−^ T cells displayed a diminished Th17 skewing *in vitro* (Figure 6E). In summary, TYK2^P^ plays a role in IL-23 signaling mostly likely contributing to the observed decrease in Tfh-17 cells in subjects expressing the protective variant.

### *Tyk2^*P*^* Mice Are Protected From EAE and Exhibit Reduced Numbers of IFN-γ^+^/IL-17^+^ Pathogenic CD4^+^ T Cells

**Figure 7:**
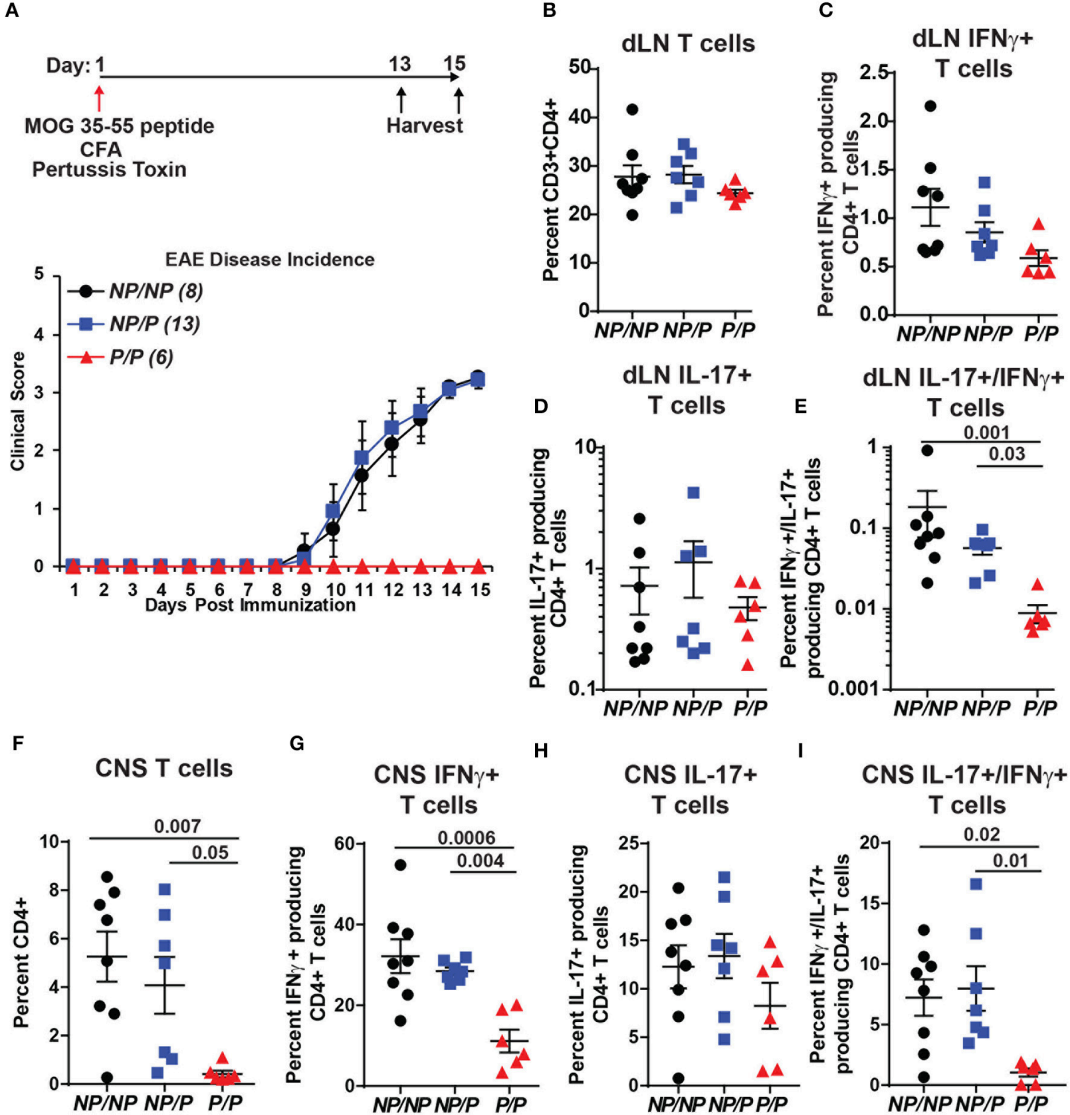
*Tyk*2^*P*^ variant mice are protected in a murine model of multiple sclerosis. **(A)** (*Upper*) Schematic of experimental design for induction of EAE by immunization with MOG peptide. *Tyk2*^*NP*/*NP*^(*n* = 8), *Tyk2*^*NP*/*P*^(*n* = 13), and *Tyk2*^*P*/*P*^(*n* = 6) mice were immunized (red arrow) and evaluated clinically for 15 days. (*Lower*) EAE clinical score was determined as per methods (1 = Tail limp, 2 = 1 hind leg paralyzed, 3 = 2 hind legs paralyzed, 4 = front leg paralyzed). **(B–G)** Tissues were collected at Day 13 or Day 15 post-immunization and T lymphocyte populations were evaluated using flow cytometry. **(B–D)** Frequency of T cells within the draining lymph nodes (dLN) showing: **(B)** total T cells; **(C)** IFN-γ^+^ T cells **(D)** IL-17^+^ T cells, and **(E)** IL-17^+^/IFN-γ^+^ double positive T cells **(F–I)**. Frequency of T cells within the central nervous system (CNS) showing: **(F)** total T cells; **(G)** IFN-γ^+^ T cells; **(H)**, IL-17^+^ T cells; and **(I)** IL-17^+^/IFN-γ^+^ T cells. **(B–I)**; Each symbol represents an individual animal; small horizontal lines indicate the mean (± s.e.m.). Flow cytometry data shown are from 2 independent experiments including: *Tyk2*^*NP*/*NP*^ (*n* = 7) *Tyk2*^*NP*/*P*^ (*n* = 7) and *Tyk2*^*P*/*P*^ (*n* = 6) animals. **(C–I)** Data shown are combined from cells collected on day 13 or 15. Statistical analysis was performed using a two-way ANOVA with Tukey's multiple comparisons test.

*TYK2*^*P*^ has also been associated with protection in MS (31). We used a murine model of MS, experimental autoimmune encephalomyelitis (EAE), to test the role of *Tyk2*^*P*^ in modulating disease. As shown schematically in Figure 7A, control (*Tyk2*^*NP*/*NP*^), heterozygous *Tyk2*^*NP*/*P*^ and homozygous *Tyk2*^*P*/*P*^ mice were immunized with MOG peptide in complete Freund's adjuvant (CFA) and also treated with pertussis toxin to increase permeability of the blood brain barrier. While both control and heterozygous *Tyk2*^*NP*/*P*^ animals developed disease manifestations beginning at ~10 days post-immunization, mice expressing *Tyk2*^*P*/^^P^ were completely protected from EAE (Figure 7A, lower panel). Both Th1 and Th17 cells have been shown to be important for EAE disease development (54). Notably, the proportion of draining LN T cells expressing IL-17^+^ was similar in *Tyk2*^*NP*/*NP*^, *Tyk2*^*NP*/*P*^ and *Tyk2*^*P*/*P*^ animals and there was only a trend toward a reduced proportion of IFN-γ^+^ cells in *Tyk2*^*P*/*P*^ animals (Figures 7B–E). In contrast, the proportion of double-positive IFN-γ^+^/IL-17^+^ pathogenic CD4^+^ T cells was specifically decreased in *Tyk2*^*P*/*P*^ mice (Figure 7E). The number of CD4^+^ T infiltrating the central nervous system (CNS) was markedly reduced in *Tyk2*^*P*/*P*^ mice and included reduction in both IFN-γ^+^ or IFN-γ^+^/IL-17^+^ double positive T cells (Figures 7F–I). Altogether, these data demonstrate that *Tyk2*^*P*^ protects from EAE by decreasing pathogenic CD4^+^ T cells which depend on both IL-12 and IL-17 signaling to promote disease development.

## Discussion

While *TYK2*^*P*^ has been shown to be a hypomorphic allele, its protective role in autoimmunity still remains largely unexplored. Here we show that *TYK2*^*P*^ limits signaling in response to IL-12, IL-23, and IFN I cytokines. Despite these cytokine defects, *Tyk2*^*P*^ mice were not protected in two independent lupus models and exhibited no difference in the response toward two different T dependent immunization models. Yet healthy individuals expressing *TYK2*^*P*^ displayed diminished Tfh and switched memory B cells, and homozygous *Tyk2*^*P*^ mice were fully protected in a murine model of MS. Our findings highlight the complexity of the cytokine milieu that regulate immune responses in both man and mouse, and the likely requirement for concurrent alterations in multiple cytokine signals in order for this variant to manifest a disease protective phenotype.

### Functional Role of *TYK2^*P*^* in Cytokine Signaling

In our murine model, we found a deficiency in IL-12 induced pSTAT3 in homozygous *Tyk2*^*P*^ expressing CD4^+^ T cells. This was complimentary to a recent study that developed a similar mouse model of *Tyk2*^*P*1104*A*^ and showed decreased IL-12 induced pSTAT4 (33). Similar to the murine data, Dendrou et al. found diminished IL-12 induced pSTAT4 in human CD4^+^ T cells expressing *TYK2*^*P*/*P*^ compared to *TYK2*^*NP*/*NP*^ T cells (33). In contrast, we did not identify differences in pSTAT3 or pSTAT4 following IL-12 stimulation in homozygous non-protective vs. heterozygous protective individuals. Our findings are consistent with a recent data set comparing *TYK2*^*NP*/*NP*^ to *TYK2*^*NP*/*P*^ participants (55). Differences between our studies likely reflect the large number of homozygous *TYK2*^*P*^ individuals (7 vs. 2) studied by Dendrou et al. and/or differences in stimulation conditions. Further, we found that IL-23 signaling and IL-17^+^ cells were decreased in murine homozygous *Tyk2*^*P*^ CD4^+^ Th17 populations consistent with previous mouse and human data (33). Lastly, we demonstrate that TYK2^P^ also limits type I interferon signaling in humans, and in the *Tyk2*^*P*^ murine model (data not shown) as observed in human *TYK2*^*P*^ T cells (33). Of note, while *Tyk2*^*NP*/*NP*^ and *Tyk2*^*NP*/*P*^ T and B cells did not exhibit statistically significant differences in IL-12 mediated signals (Figures 2A–D), in each assay, *Tyk2*^*NP*/*P*^ cells showed a slight decrease in pSTAT3 and Th skewing (and in IL-12 triggered B cell IL-6 production) compared to *Tyk2*^*NP*/*NP*^ cells suggesting a potential dose-dependent effect on *in vitro* IL-12 signaling. The findings mimicked the impact of heterozygous dosage of the protective variant in human cells in various settings. Taken together, our observations support the conclusion that TYK2^P^ exerts an allele-dose dependent limiting effect on *in vitro* responses to IL-12, IL-23, and IFN I signaling.

Individuals that are *TYK2*-deficient manifest impaired cytokine responses to IL-12, IL-23, IFN-α, and IL-10 (21). Moreover, these patients exhibit an increased risk for mycobacterial and viral infections (21). Consistent with this phenotype, *TYK2*^*P*^ individuals also exhibit signaling defects in IL-12, IL-23, and IFN-α. However, based upon the clinical data within our biorepository, the small number of homozygous protective variant-expressing subjects have not displayed increased infections similar to the *TYK2*-deficient patients (21) and other studies to date also have not reported an increase in infectious risk for such individuals; suggesting that larger populations studies are likely required to address this question (33). Differences between complete *TYK2* deficiency versus a hypomorphic allele may reflect retention of a protein scaffold function. This idea may also be consistent with observations that individuals or mice heterozygous for the protective allele exhibit subtle alterations in lymphocyte subsets and signaling activity, implying a possible dominant negative effect of the protective allele. Studies have also linked loss of TYK2 expression to altered stability of STAT proteins in murine cells and TYK2 associated receptor surface expression on human cells (20, 21, 23, 33). Similar findings have not been previously reported or observed in our *Tyk2*^*P*^ murine model (data not shown). TYK2^P^ expression was shown not to affect IFNAR surface expression (Figures 5C,D) and IL-12R (33). More work is needed to fully elucidate the TYK2 interactome in various cell lineages and its impact(s) in modulation of cytokine signaling.

Dysregulation of the IL-12, IL-23, or IFN signaling pathways may also contribute to SLE disease (3, 15, 16). However, signaling molecules within these pathways seem to compensate for each other. Hence, there is the need for multiple aberrant pathways to lead to complex autoimmune diseases such as SLE. There is evidence that these pathways are on a fine axis. When one is dysregulated, it throws off the balance of the other pathways leading to further abnormal signaling, irregular activation and ultimately autoimmune disease. One example of this is deficiency in STAT3 which causes a decrease in Tfh and GC B cells, leading Th cells to take on the Th1-like phenotype. However, in STAT3 deficient cells the normal populations are rescued when IFNAR is blocked (18). Together these cytokine pathways are dependent on one another *in vivo* and must be studied collectively to get a complete picture of how such signals contribute to disease.

### TYK2^P^ and T Helper Subsets

IL-12 and IL-23 represent critical cytokines for generation of Th1 and Th17 cells, respectively. Herein we show that IL-12 and IL-23 signaling and their respective Th subsets are diminished in an *in vitro* setting when TYK2^P^ is expressed. Importantly, these cytokines are also involved in Tfh cell generation. We show for the first time that Tfh cells, specifically the Tfh-17 cell subset which has superior ability to provide B cell help (56), are reduced in healthy human subjects with the *TYK2*^*P*^ allele. Further, we show that naïve murine *Tyk2*^*P*^ CD4^+^ T cells exhibit a defect in *in vitro* Tfh generation. Consistent with these findings, individuals lacking *IL-12R*β*1* exhibit diminished circulating memory Tfh and memory B cells (57). IL-23 also signals through STAT3 and can to contribute to Tfh generation (13). Our combined observations support a model wherein combined reduction in IL-12 and IL-23 signals leads to a reduced number of Tfh-17 cells in healthy *TYK2*^*P*^ donors. Thus, TYK2^P^ is a critical regulator for Tfh populations by reducing IL-12 and IL-23 signaling cascades.

Herein we also found healthy individuals expressing TYK2^P^ to have diminished switched memory B cells. This is consistent with *IL-12R*β*1* deficient subjects who exhibited both reduced switched and unswitched memory B cells (57). This reduction is most likely due to defective GC responses from diminished IL-12 signaling in T cells. IL-12 is an efficient inducer of IL-21 production from Tfh cells, a cytokine critical for the activation of human GC B cells (14, 58). Additionally, *IL-12R*β*1-, TYK2-*, or *STAT3*-deficient CD4^+^ T cells display reduced IL-12 induced IL-21 production *in vitro* (12). However, *Tyk2*^*P*^ mice did not display any differences in GC responses post-immunization. Further investigation is needed to assess GC formation and its link to memory B cells in *Tyk2*^*P*^ mice.

### TYK2^P^ in Autoimmune Disease

*TYK2*^*P*^ has been associated with protection from MS (31) and *Tyk2*^−/−^ mice are fully protected from EAE (26). In our study, we found that homozygous *Tyk2*^*P*/*P*^ mice are completely protected from EAE. Infiltrating T cells within the CNS were markedly reduced in *Tyk2*^*P*/*P*^ mice and protection correlated most strongly with a reduction in double-positive IFN-γ^+^/IL-17^+^ CD4^+^ T cells within both the draining lymph nodes and the CNS. Of note, consistent with the partial *in vitro* phenotype in response to cytokine stimulation, heterozygous *Tyk2*^*NP*/*P*^ mice exhibited a trend toward reduced single IFN-γ^+^ and double-positive IFN-γ^+^/IL-17^+^ CD4^+^ T cells in the draining lymph nodes. However, heterozygous animals were not protected from EAE *in vivo*. Our combined findings are consistent with and expand upon previous data from Dendrou et al. (33). Both the IL-12 and IL-23 signaling programs contribute to EAE disease (59). Protection for EAE in *Tyk2*^*P*/*P*^ mice aligns with the reduced IL-12 and IL-23 signaling and reduced Th1 and Th17 *in vitro* skewing described above. MS patients also exhibit populations of Tfh-1 and Tfh-17 cells and the relative proportions of these effectors varies among MS cohorts, with IL-23 signaling playing a more dominant role in some subjects(3, 60). More work is required to determine whether protection from MS in *TYK2*^*P*^ carriers might be predicted based upon the proportion of Tfh-17 cells and/or dual-positive IFN-γ^+^/IL-17^+^ CD4^+^ effector T cells.

In contrast to the EAE data, we show that *Tyk2*^*P*^ does not shield mice from autoantibody production and disease progression in two separate lupus models even though GWAS has linked this variant to protection from SLE (28–30, 33). SLE is a heterogeneous disorder that reflects both variable genetic and environmental contributions. The lack of protection observed in our studies may reflect the specific disease models studied. We observed no impact of *Tyk2*^*P*^ in the BM12 adoptive transfer lupus model where autoantibody production is driven by self-reactive T cell triggered autoimmune GC responses that are characterized by expanded Tfh populations. Despite our findings of altered Tfh and memory B cell populations in healthy *TYK2*^*P*^ subjects, we did not observe alterations in Tfh or autoantibody generation in this model. We also showed no impact of *Tyk2*^*P*^ in the *WAS* B cell chimera lupus model. This latter model leads to spontaneous autoimmune GC responses driven by altered B cell receptor (BCR) and TLR7 signaling. Autoimmune GC production is also dependent upon B cell intrinsic antigen presenting cell (APC) activity, IFNγ1-R1 signaling, and IL-6 production (46, 51). Surprisingly, in the current study, we also show that the *WAS* chimera model is not impacted by global *Il12rb2* deficiency and our previous work has shown that B cell-intrinsic IFNAR is also dispensable for lupus development in this model (51). Thus, two key programs modulated by *Tyk2*^*P*^ play a limited role in this model. As noted above, TYK2^P^ can function to limit IFN I signaling. IFN I signaling is increased in a subset of SLE subjects and IFN I blockade has provided partial benefit in some patients (61–63). Thus, the potential protective impact of *Tyk2*^*P*^ may be most relevant in lupus models that are driven or accelerated by an altered IFN I program. Future studies using co-modeling with other relevant SLE GWAS risk alleles, including the common *IFIH*1 risk variant (37), may provide insight into the impact of TYK2^P^ in SLE disease pathogenesis.

*TYK2*^*A*1104^ allele is a rare variant at ~2.7% overall allelic frequency (64). Thus, the association with protection in multiple autoimmune disorders is predominantly within heterozygous individuals. As noted above, while we observed alterations in key lymphocyte populations in healthy subjects with the protective allele, we observed only trends toward reduced signaling activity using heterozygous *Tyk2*^*NP*/*P*^ murine and human cells in our *in vitro* studies. Disease protection *in vivo*, when present, was only evident in *Tyk2*^*P*/*P*^ animals. The requirement for homozygous TYK2^P^ expression to manifest differences in our assays suggests that protection likely involves a more complex process than simply altering a single cytokine program. Instead, the variant appears to provide protection by modestly altering multiple pathways, thereby subtly diminishing immune responses that lead to autoimmunity. This complex role for TYK2^P^ in protection from autoimmune pathogenesis highlights the value of our combinatorial studies using both murine models and healthy human subjects to assess its impact on human disease. Whether protection primarily reflects diminished Tfh cell populations or another cell type remains to be fully defined the ability of TYK2 to impact a subset of key cytokine pathways highlights its potential utility as a therapeutic target. Consistent with this concept, recent findings using an oral TYK2 inhibitor have demonstrated beneficial effects in treatment of adult subjects with psoriasis (65). Taken together, our findings suggest that targeting TYK2 kinase activity may provide a relatively broad therapeutic window for protection from autoimmune disease while limiting the potential risk for immunosuppression.

## Data Availability Statement

The data that support the findings of this study are available for the corresponding author upon request.

## Author Contributions

JG designed and performed experiments, analyzed data, and wrote the manuscript. CH designed and performed experiments, analyzed data, and edited manuscript. MK, TA, EA, CC, SW, KT, AE, SK, MH, and MO developed required models/strains or reagents and/or performed experiments and/or edited manuscript. SWJ designed and interpreted WASp mouse studies. KC genotyped human subjects, interpreted data, and edited manuscript. JHB and DJR conceived and supervised the study, interpreted data, and edited the manuscript.

### Conflict of Interest Statement

The authors declare that the research was conducted in the absence of any commercial or financial relationships that could be construed as a potential conflict of interest.
