# Supplementary material for: The TYK2-P1104A Autoimmune Protective Variant Limits Coordinate Signals Required to Generate Specialized T Cell Subsets
Source: Front Immunol. 2019 Jan 25;10:44. doi: 10.3389/fimmu.2019.00044 (PMC6355696; doi:10.3389/fimmu.2019.00044)
Supplement: Supplementary file 1 [file Data_Sheet_1.pdf]

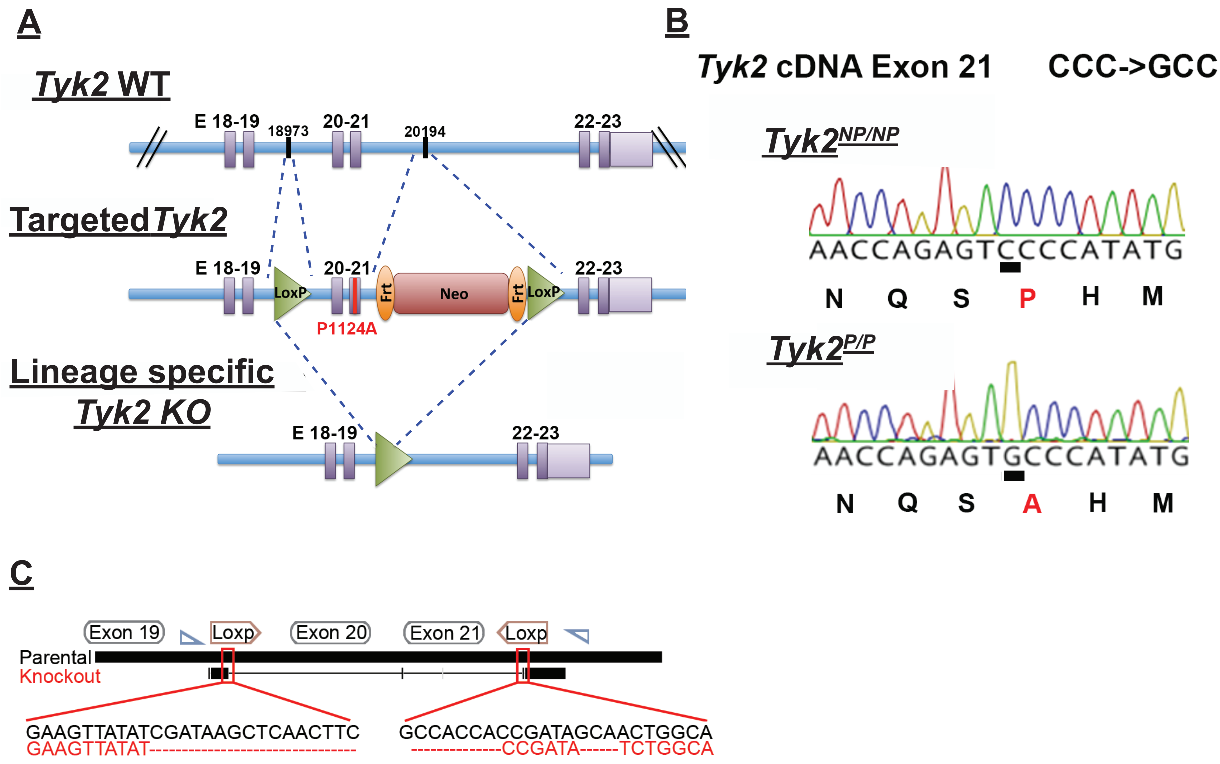

Supplementary Figure 1. Generation of *Tyk2*^{P1124A} and *Tyk2*$^{-/-}$ murine models.
(**A**) Strategy for generating the *Tyk2*^{P} knock-in mice with introduction of the point mutation within exon 21 of *Tyk2* designed to introduce the protective variant allele. Also shown are location of neomycin cassette with flanking FRT sites and the location of *LoxP* sites introduced in order to permit generation of lineage-specific *Tyk2* deletion via intercrossing with cre-expressing strains. (**B**) DNA sequencing reaction showing the *Tyk2* coding change from **C**CC (Pro) to **G**CC (Ala) in homologous knock-in (*Tyk2*$^{P/P}$) mice. (**C**) DNA sequencing reaction showing the *Tyk2* coding change at *LoxP* sites *Tyk2*$^{P/P}$ (Parental) to *Tyk2*$^{-/-}$ (Knockout) introduced by crossing the knock-in mice to CMV-cre expressing mice to create a global knockout within the same genetic background.

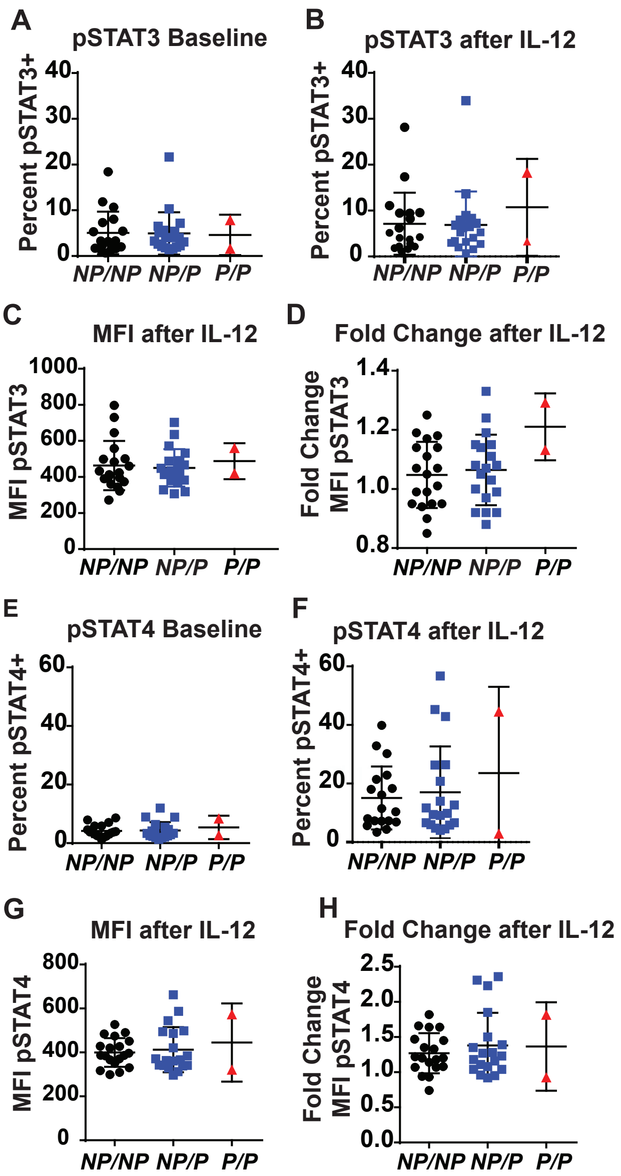

<u>Supplementary Figure 2.</u> TYK2^{P} does not alter IL-12 signaling in human CD4 memory T cells. Preactivated PBMC from subjects with *TYK2$^{NP/NP}$* (*NP/NP*), *TYK2$^{NP/P}$* (*NP/P*), and *TYK2$^{P/P}$* (*P/P*) were thawed and stimulated with 2.5 ng/ml of IL-12 or left unstimulated for 30 minutes. CD4^{+}CD45RA^{-} memory T cells were assessed by flow cytometry for: (**A**) proportion of pSTAT3 at baseline and (**B**) in response to IL-12. (**C**) pSTAT3 mean fluorescence intensity (MFI) after IL-12. (**D**) Fold change of pSTAT3 MFI from baseline in response to IL-12 stimulation. (**E**) Frequency of pSTAT4 at baseline and (**F**) and in response to IL-12. (**G**) pSTAT4 MFI after IL-12. (**H**) Fold change of pSTAT4 MFI from baseline in response to IL-12 stimulation. Each symbol represents an individual donor (**A-H**); small horizontal lines indicate the mean (± s.d.). Data from a combined total of n=19 *TYK2$^{NP/NP}$* donors, n=19 *TYK2$^{NP/P}$* donors, and n=2 *TYK2$^{P/P}$* donors.

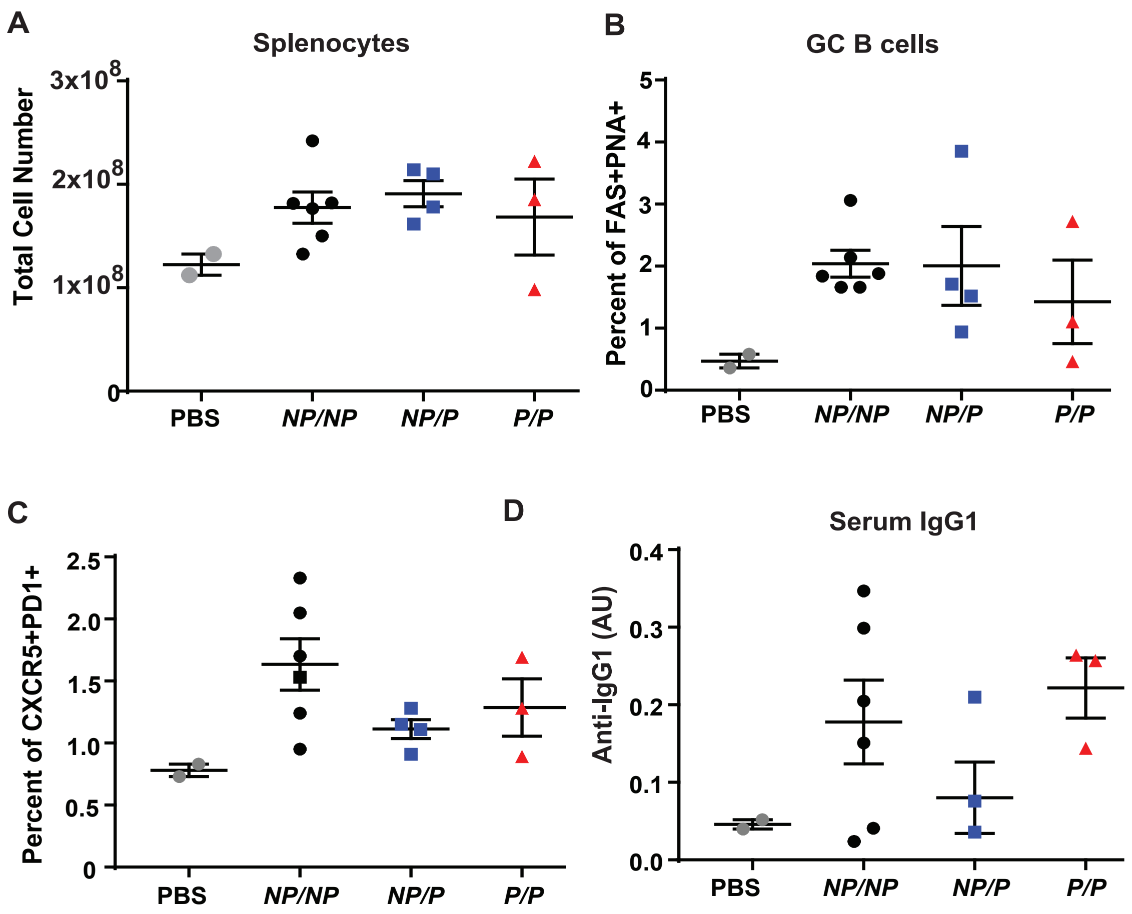

<u>Supplementary Figure 3</u>. Tfh and GC B cell formation *in vivo* in response to immunization with sheep red blood cells is not impacted by *Tyk2^{P}* expression. (**A-C**).*Tyk2$^{NP/NP}$* (*NP/NP*), *Tyk2$^{NP/P}$* (*NP/P*), *Tyk2$^{P/P}$* (*P/P*) or *Tyk2$^{-/-}$* (*KO*) mice were immunized i.p. with 20% by volume of sheep red blood cells (SRBC) or PBS. *Tyk2$^{NP/NP}$* and *Tyk2$^{NP/P}$* mice were used for PBS controls. Splenocytes and serum were collected at Day 5 post-immunization and analyzed for: (**A**) Total splenocyte cell numbers; (**B**) frequency of GC B cells (B220+FAS+PNA+ cells); (**C**) frequency of Tfh cells ($CD4^{+}CXCR5^{+}PD1^{+}$ cells); (**D**) ELISA for IgG1 antibody (dilution of 31250). Each symbol represents an individual biological replicate; small horizontal lines indicate the mean (± s.e.m.). Data shown are from one of two independent experiments. Statistical analysis was performed using one-way ANOVA with Tukey's multiple comparisons test (**A-C**).

Supplementary Table 1

**Experiment**	**Control**		**Protective**		**Figure**
	**Protein**	**Genotype Nomenclature**	**Protein**	**Genotype Nomenclature**	
Primary Human PBMCs	TYK2$^{Val362/Phe362}$TYK2$^{Pro1104/Pro1104}$	TYK2$^{NP/NP}$	TYK2$^{Val362/Phe362}$TYK2$^{Pro1104/Ala1104}$	TYK2$^{NP/P}$	Figure 1
			TYK2$^{Phe362/Phe362}$TYK2$^{Ala1104/Ala1104}$	TYK2$^{P/P}$	
In vitro Murine cells	TYK2$^{Pro1124/Pro1124}$	TYK2$^{NP/NP}$	TYK2$^{Pro1124/Ala1124}$	TYK2$^{NP/P}$	Figure 2
			TYK2$^{Ala1124/Ala1124}$	TYK2$^{P/P}$	
			TYK2$^{-/-}$	KO	
In vivo Murine model	TYK2$^{Pro1124/Pro1124}$	TYK2$^{NP/NP}$	TYK2$^{Pro1124/Ala1124}$	TYK2$^{NP/P}$	Figure 3
	TYK2$^{Pro1124/Pro1124}$	PBS	TYK2$^{Ala1124/Ala1124}$	TYK2$^{P/P}$	
In vivo Murine model	TYK2$^{Pro1124/Pro1124}$	TYK2$^{NP/NP}$	TYK2$^{Pro1124/Ala1124}$	TYK2$^{NP/P}$	Figure 4A-C
			TYK2$^{Ala1124/Ala1124}$	TYK2$^{P/P}$	
In vivo Murine model	TYK2$^{Pro1124/Pro1124}$	TYK2$^{NP/NP}$	TYK2$^{Pro1124/Ala1124}$	TYK2$^{NP/P}$	Figure 4E-H
	TYK2$^{Pro1124/Pro1124}$IL-12R$^{-/-}$	IL-12R$^{-/-}$	TYK2$^{Ala1124/Ala1124}$	TYK2$^{P/P}$	
Primary Human PBMCs	TYK2$^{Val362/Phe362}$TYK2$^{Pro1104/Pro1104}$	TYK2$^{NP/NP}$	TYK2$^{Val362/Phe362}$TYK2$^{Pro1104/Ala1104}$	TYK2$^{NP/P}$	Figure 5
Primary Human PBMCs	TYK2$^{Val362/Phe362}$TYK2$^{Pro1104/Pro1104}$	TYK2$^{NP/NP}$	TYK2$^{Val362/Phe362}$TYK2$^{Pro1104/Ala1104}$	TYK2$^{NP/P}$	Figure 6A-C
			TYK2$^{Phe362/Phe362}$TYK2$^{Ala1104/Ala1104}$	TYK2$^{P/P}$	
In vitro Murine cells	TYK2$^{Pro1124/Pro1124}$	TYK2$^{NP/NP}$	TYK2$^{Pro1124/Ala1124}$	TYK2$^{NP/P}$	Figure 6D-E
			TYK2$^{Ala1124/Ala1124}$	TYK2$^{P/P}$	
			TYK2$^{-/-}$	KO	
In vivo Murine model	TYK2$^{Pro1124/Pro1124}$	TYK2$^{NP/NP}$	TYK2$^{Pro1124/Ala1124}$	TYK2$^{NP/P}$	Figure 7
			TYK2$^{Ala1124/Ala1124}$	TYK2$^{P/P}$	
